# Transcriptional bursting in *Drosophila* development: Stochastic dynamics of *eve* stripe 2 expression

**DOI:** 10.1371/journal.pone.0176228

**Published:** 2017-04-24

**Authors:** David M. Holloway, Alexander V. Spirov

**Affiliations:** 1 Mathematics Department, British Columbia Institute of Technology, Burnaby, B.C., Canada; 2 Biology Department, University of Victoria, Victoria, B.C., Canada; 3 Computer Science, and Center of Excellence in Wireless and Information Technology, State University of New York, Stony Brook, New York, United States of America; 4 Sechenov Institute of Evolutionary Physiology and Biochemistry, St. Petersburg, Russia; Oxford Brookes University, UNITED KINGDOM

## Abstract

Anterior-posterior (AP) body segmentation of the fruit fly (*Drosophila*) is first seen in the 7-stripe spatial expression patterns of the pair-rule genes, which regulate downstream genes determining specific segment identities. Regulation of pair-rule expression has been extensively studied for the *even-skipped* (*eve*) gene. Recent live imaging, of a reporter for the 2^nd^ *eve* stripe, has demonstrated the stochastic nature of this process, with ‘bursts’ in the number of RNA transcripts being made over time. We developed a stochastic model of the spatial and temporal expression of *eve* stripe 2 (binding by transcriptional activators (Bicoid and Hunchback proteins) and repressors (Giant and Krüppel proteins), transcriptional initiation and termination; with all rate parameters constrained by features of the experimental data) in order to analyze the noisy experimental time series and test hypotheses for how *eve* transcription is regulated. These include whether *eve* transcription is simply OFF or ON, with a single ON rate, or whether it proceeds by a more complex mechanism, with multiple ON rates. We find that both mechanisms can produce long (multi-minute) RNA bursts, but that the short-time (minute-to-minute) statistics of the data is indicative of *eve* being transcribed with at least two distinct ON rates, consistent with data on the joint activation of *eve* by Bicoid and Hunchback. We also predict distinct statistical signatures for cases in which *eve* is repressed (e.g. along the edges of the stripe) vs. cases in which activation is reduced (e.g. by mutagenesis of transcription factor binding sites). Fundamental developmental processes such as gene transcription are intrinsically noisy; our approach presents a new way to quantify and analyze time series data during developmental patterning in order to understand regulatory mechanisms and how they propagate noise and impact embryonic robustness.

## Introduction

**Fig 1:**
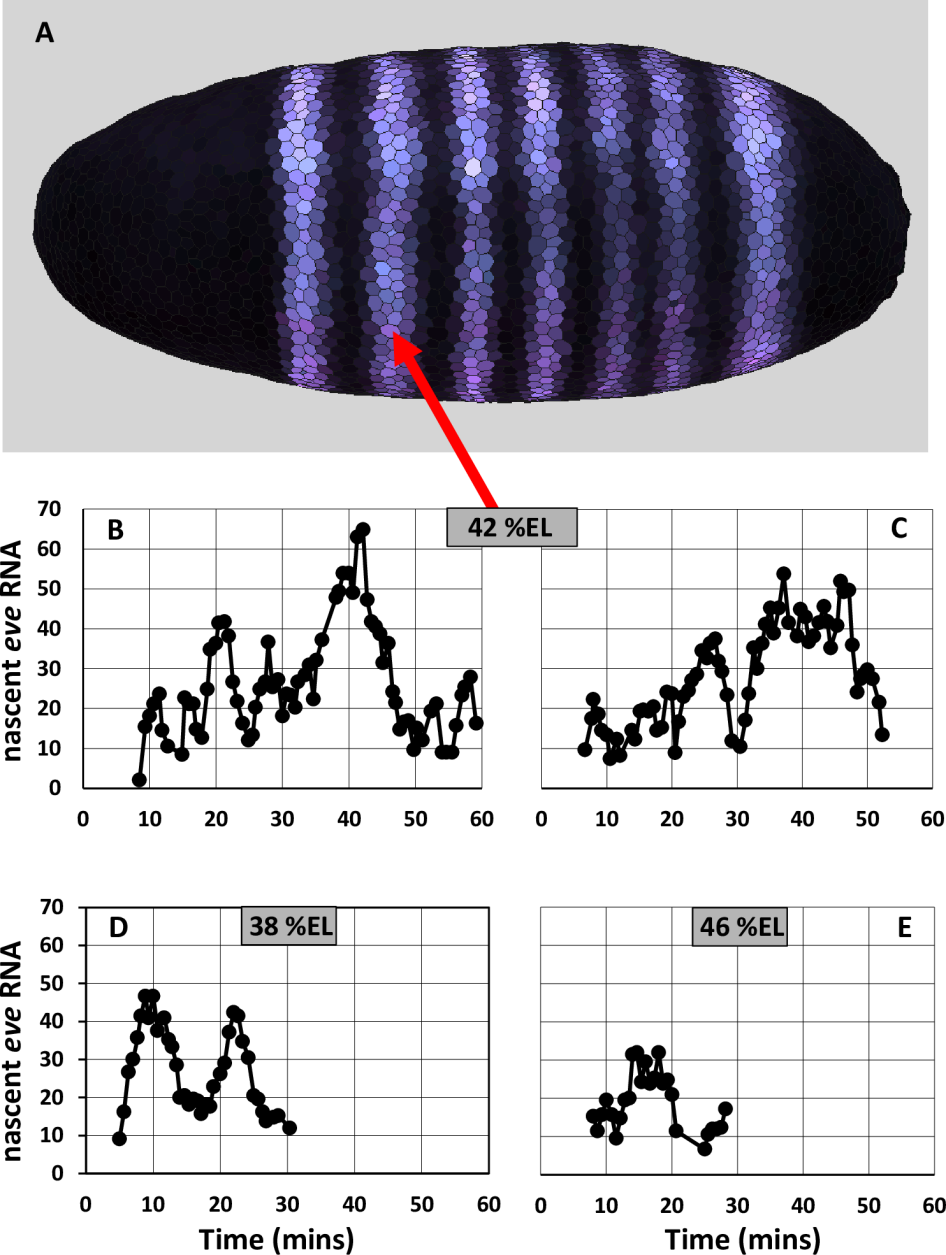
mRNA expression for the pair-rule gene *even-skipped* (*eve*) in *Drosophila*. (A) Spatially, *eve* (purple) is expressed in 7 stripes orthogonal to the anterior-posterior (AP) axis, each 5–10%EL (percent embryo length) wide. Anterior is to the left. Data from the BDTNP database (http://bdtnp.lbl.gov/Fly-Net/), nuclear and cytoplasmic staining for *eve* mRNA in embryo 11081-02jn060-06 at 40% membrane invagination (mid nuclear cleavage cycle 14), projected onto a canonical embryo geometry. Image created with *PointCloudXplore*. Expression at stripe 2, centered at approximately 42%EL from the anterior pole (red arrow), can be driven by the minimal stripe element (MSE). (B—E) Time series of the number of nascent *eve* transcripts in stripe 2 show stochastic gene expression at per minute resolution. (B, C) Time series from two individual nuclei at stripe center (approx. 42%EL), data from Fig 4A and S2B Fig respectively of [4]. (D) Time series from a nucleus on the anterior edge of stripe 2 (approx. 38%EL), data from S2ig of [4]. (E) Time series from a nucleus at the posterior edge of stripe 2 (approx. 46%EL), data from S2D Fig of [4].

Segmentation of the early fruit fly (*Drosophila*) embryo has long been a model system for studying the genetic control of spatial patterning in development [1–3]. Along the anterior-posterior (AP) axis, broadly distributed maternally derived transcription factors (TFs) and zygotically expressed gap gene TFs combine to regulate the finer spatial scale expression of the pair-rule genes (Fig 1A). These narrow stripes of pair-rule expression are the first manifestation of the segmented insect body plan, since pair-rule encoded TFs subsequently regulate segment identity genes which control specific differentiation pathways for particular segments.

The expression and regulation of the pair-rule gene *even-skipped* (*eve*) has been extensively studied for many years [5]. *eve* was the first pair-rule gene in which it was shown that individual stripes can be regulated by specific cis-regulatory sequences of the DNA. Reporter constructs driven by a 1.7 kb sequence upstream of the *eve* coding region express strongly at the 2^nd^ *eve* stripe (*eve2*) position (Fig 1A, red arrow) and more weakly at the 7^th^ stripe position [6,7]. It was subsequently found that a 480 bp sequence within this, the minimal stripe element (MSE), could drive strong expression exclusively at the *eve2* position [8]. The MSE has binding sites (BSs) for the transcriptional activators Bicoid (Bcd) and Hunchback (Hb), and for the repressors Giant (Gt) and Krüppel (Kr) [8–10]. Bcd is maternally derived and forms an early anterior-high concentration gradient. Zygotically expressed patterns of the gap gene TFs Hb, Gt and Kr form slightly later. By the time the *eve* stripes form at nuclear cleavage cycle (nc) 14, Hb is high throughout the anterior half of the embryo, while Gt and Kr are localized to domains about 20%EL (percent embryo length) wide. The anterior Gt domain is centered at approx. 28%EL (all distances from the anterior pole) and Kr is centered at approx. 53%EL (FlyEx, http://urchin.spbcas.ru/flyex/ [11]). Bcd and Hb activate *eve* through the anterior of the embryo, and *eve* stripe 2 forms in the trough between the Gt and Kr repressor domains. Deterministic modeling of *eve* transcription (using experimental spatial patterns for the TFs) has shown that the BSs for Bcd, Hb, Gt and Kr in the 480 bp MSE are sufficient to generate the spatial and temporal characteristics of stripe 2 expression; and that flanking BSs in the full 1.7 kb sequence contribute to weak stripe 7 expression, but have negligible effect on stripe 2 expression [12].

Bothma et al. [4] recently developed a system to measure *eve2* expression in live embryos, using the 1.7kb cis-regulatory sequence coupled to a reporter gene which forms MS2 loops in the transcribing RNA, to which GFP (green fluorescent protein) binds. This technique gives fluorescent signal proportional to the number of RNA molecules in the act of being transcribed (i.e. nascent transcripts), with signal lost as transcription is completed (releasing a free mRNA molecule). The live technique was pioneered on *hb* expression [13–15], which appears relatively steady. *eve2* transcription, by contrast, is highly stochastic, with characteristic ‘bursts’ in number of nascent transcripts. Fig 1B–1E show time series for individual nuclei in the stripe 2 domain over nc 14, with ‘burst’ peaks of up to 50–60 nascent transcripts interspersed with troughs when only about 10–20 transcripts are being made. The number of nascent transcripts is lower at the stripe edges where Gt and Kr repression play a stronger role. Expression is stronger 4%EL to the anterior of the stripe center (at 38%EL; Fig 1D) under moderate Gt repression, than 4%EL to the posterior (at 46%EL; Fig 1E) under stronger Kr repression (relative to the Bcd and Hb activation). Bursting in gene expression has been studied for some time in single cells (e.g. [16–20]), leading to a greater understanding of the inherent stochasticity of transcription and translation, but the *eve2* time series are the first from a developing spatial pattern in a metazoan embryo. This offers a unique view into transcriptional noise in a complex developmental process, and an opportunity to quantify how regulatory mechanisms affect expression variability, both spatially and temporally.

Traditionally, bursting in transcription has been approached through a simple ON-OFF model, with stochastic switching between the OFF state and a single ON-state with a characteristic transcription rate (e.g. [21]). New experiments and modeling are indicating that multi-state transcription, with multiple ON-states each with a characteristic rate, may play a role in many cases. Single ON-state vs. multiple ON-state transcription in single cells was recently reviewed in [22]; see also [23] regarding the effect of variation in initiation rates, and [24] on the noise effects of multi-step initiation. For *eve2*, Bothma et al. [4] suggested that the different burst peak heights (in Fig 1B–1E) reflected multiple ON-states. However, classifying peaks in a noisy time series can be highly sensitive to the technique for estimating burst duration and height, adding uncertainty to any inference between bursts and the underlying transcriptional mechanism. Specifically, establishment of a multiple ON-state mechanism should rule out whether the stochastic dynamics of a simple ON-OFF mechanism can generate the characteristics of the observed time series.

We introduce a stochastic modeling approach to directly evaluate the relation between the *eve2* time series and the underlying transcriptional mechanism. The model is constrained by spatial and temporal characteristics of the data, and does not require estimation of burst peak size. We find that the broad characteristics of the data, in particular the multi-minute bursts in nascent transcript number, can be generated both by a simple ON-OFF mechanism and by a two-ON-state model: i.e., the mechanisms cannot be distinguished at coarse timescales. However, at the finest resolution of the data (per-minute), the two-ON-state model more closely fits statistical characteristics of the experimental time series than the simple ON-OFF mechanism. This suggests that the different regulation of *eve2* by Bcd and Hb indicated by earlier experiments [8,25] may be evident in time series at suitably fine resolution. We predict that increased repression, such as at the stripe edges, should show different statistical characteristics than decreased activation, such as with mutagenesis of the Hb BS [8,25]. As such fine scale temporal expression data becomes more available, our approach presents a technique for identifying different components of a gene’s regulatory mechanism from noisy time series and studying how different regulatory dynamics contribute to gene expression noise and developmental robustness.

## Model and methods

### Model specification

Experiments [8] and modelling [12] show that the cis-regulatory 480 bp MSE can drive spatially and temporally normal *eve* expression at the stripe 2 position. We have developed a stochastic model for *eve* stripe 2 transcription in which the rate of transcriptional initiation depends on the bound-state of the *eve2* MSE for the TFs Bcd, Hb, Gt, and Kr. Experimental spatial patterns for these TFs serve as input to the MSE model (S1 Fig; adapted from nc14 stage T1 FlyEx data, http://urchin.spbcas.ru/flyex/, [11]). Strong BSs for these have been mapped in the MSE: 5 for Bcd, 1 for Hb, 3 for Gt, and 3 for Kr [8]. While site-directed mutagenesis shows some activation from the bcd-2 and bcd-3 BSs, the bcd-1 BS (the most 3’ in the MSE) is critical–without it there is no *eve2* expression [8]. We model Bcd regulation via this single BS. For Hb, the single BS in the model represents the single hb-3 site in the MSE. Arnosti et al. [25] showed that a single Gt BS, gt-2, was sufficient to repress the anterior border and generate a normal *eve2* stripe; the model Gt BS corresponds to this site. Kr is a weaker repressor of *eve2* than Gt [10], indicating less complex regulatory dynamics which can be sufficiently modeled through a single Kr BS.

**Fig 2:**
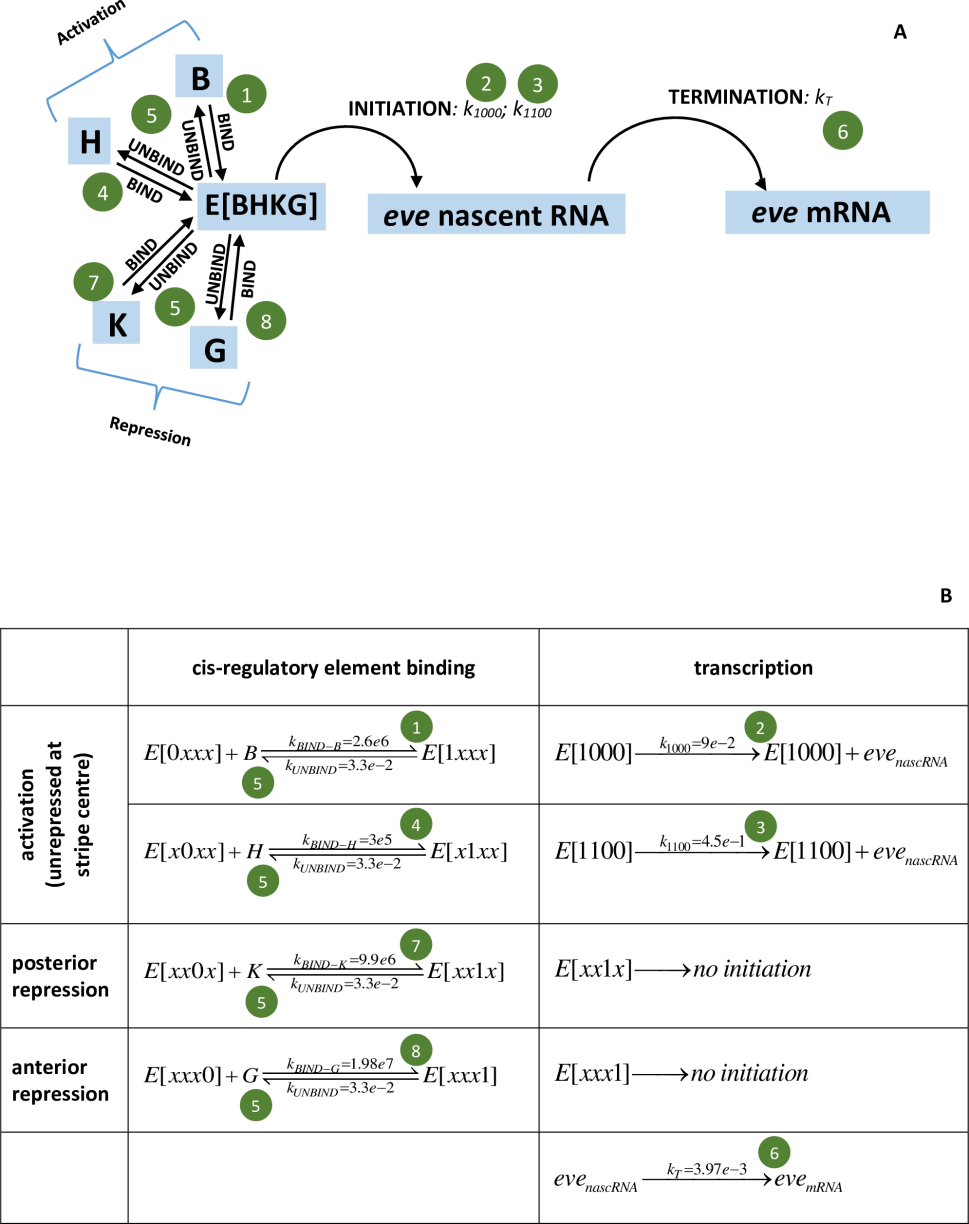
Model for *eve*2 transcriptional regulation. (A) Schematic representation; (B) representation as elementary reactions. E[BHKG] represents the *eve*2 MSE cis-regulatory region, with BSs for the activating TFs Bcd (B) and Hb (H) and for the repressing TFs Kr (K) and Gt (G). Transcription is initiated from the E[1000] state (only Bcd bound) with rate constant *k*_1000_, and from the E[1100] state (Bcd and Hb both bound, no repressors bound) with rate constant *k*_1100_. Binding of the repressors Kr or Gt blocks initiation (state E[xx1x] or E[xxx1], where x is 0 or 1). Initiation starts formation of the nascent transcript (native gene *eve*, or reporter), which can be detected by the Bothma et al. [4] technique. Termination completes transcription, freeing an mRNA into the nucleus and cytoplasm, and ending the nascent signal. The reactions (arrows in (A) and (B)) are solved stochastically by a master equation approach. The model parameters (rate constants *k*) are determined from the experimental data (green circle numbers correspond to the steps in the *Parameter estimation* subsection below). *k* units are s^-1^ for 1^st^ order reactions and M^-1^ s^-1^ for 2^nd^ order.

The model for *eve2* transcription is shown in Fig 2, with a BS each for Bcd (B), Hb (H), Kr (K), and Gt (G). The rate of transcriptional initiation depends on the bound-state of these BSs in the cis-regulatory region, E[BHKG]. The values for each BS can be 0 (unbound) or 1 (bound), e.g. E[0000] represents the *eve2* cis-regulatory region unbound by the TFs. Each black arrow in Fig 2A represents a reaction, with dynamics solved as described below. Fig 2B gives a representation of the model as elementary reactions. Transcriptional initiation requires Bcd binding (state E(1x00); x denoting either 0 or 1 for the TF); corresponding to the critical role of the bcd-1 BS in activating expression [8,25]. Mutagenesis of the hb-3 site decreases MSE expression (ibids.), indicating that Hb is a co-activator with Bcd. These results correspond to a low initiation rate, with rate constant *k*_1000_, when only Bcd is bound (the E[1000] state), and a higher initiation rate, with rate constant *k*_1100_, when both Bcd and Hb are bound (the E[1100] state). Binding of either repressor, Kr or Gt, shuts off initiation (states E[xx1x] or E[xxx1] respectively; see [10] regarding the strong repression of Bcd+Hb activation by Kr and Gt). Termination of transcription, producing free mRNA from the nascent transcript proceeds with rate constant *k*_T_.

By using a single BS for each TF, the model addresses the transcriptional effect of having a particular species of TF bound or not. It does not explicitly address TF-TF interactions or position dependent effects, though these effects can be implicit in the initiation rates for each E[BHKG] state. For example, in vivo contributions from the bcd-2 and bcd-3 BSs would be rolled into the E[1000] model initiation rate; and Hb can only act as a co-activator, with the E[1100] rate greater than the E[1000] rate, and the E[0100] rate equal to zero.

### Model solution

All reactions–binding/unbinding of TFs, transcriptional initiation and termination–are solved using the MesoRD package (http://mesord.sourceforge.net/, [26]). Deterministic solution of the model (as a system of ODEs, ordinary differential equations; 4^th^ order Runge-Kutta) is used to constrain the rate constant parameters to match experimental averages for expression levels, positions and timing. The model is then solved stochastically to tune the parameters according to the statistical features of the data (see section below). Stochastic solution uses a reaction-diffusion master equation approach with a next-subvolume queuing method to decrease solution times from a direct Gillespie algorithm [27,28]. The model is solved in subvolume units corresponding to 1 nucleus (1%EL width), each (5 μm)^3^, from 30 to 55%EL along the AP axis. See also [29,30] on the combined deterministic-stochastic approach for model development and prediction of gene expression noise. The XML file (eve2-3055.txt) specifying the model can be downloaded from http://davidhollowayresearch.weebly.com/software.html and run from the mesord command line.

### Spatial and temporal dependence

45 minute time series are simulated to match the nc14 transcription intervals measured experimentally (Fig 1B–1E; the observed initial lag of ~10 mins is not modeled). Spatial distributions of the TFs (S1 Fig) determine the spatial extent of *eve2* stripe formation. Bcd and Hb are held fixed throughout the nc14 simulation; Kr and Gt concentrations are increased ten-fold in this time (comparable to the nc13—nc14 increase [31]), to match the experimentally observed sharpening of the *eve2* stripe.

### Statistics on experimental time series

To quantify characteristics of the experimental time series to use in model parameter estimation, we calculated autocorrelation functions for both the number of nascent *eve2* transcripts and the per-minute change in number of nascent *eve2* transcripts (using *Minitab 17*). This involves calculating correlation between pairs of regularly-sampled time points so that, for example, it can be seen whether correlation exists for transcript data separated by lags of 1 minute, 2 minutes, etc. The experimental sampling rate was on the order of 1 minute, but irregular. The time series were regularized for the autocorrelation analysis by averaging data for each integer minute.

For the number of nascent transcripts at stripe-center (data in Fig 1B and 1C), there is significant autocorrelation at time lags of 1, 2, and 3 minutes (S2 Fig, red lines are 5% significance limits, i.e. zero ± t-value times standard error for autocorrelation at each time lag). Autocorrelation quantifies the transcriptional bursting in terms of the tendency for the number of transcripts to stay related for several minutes.

**Fig 3:**
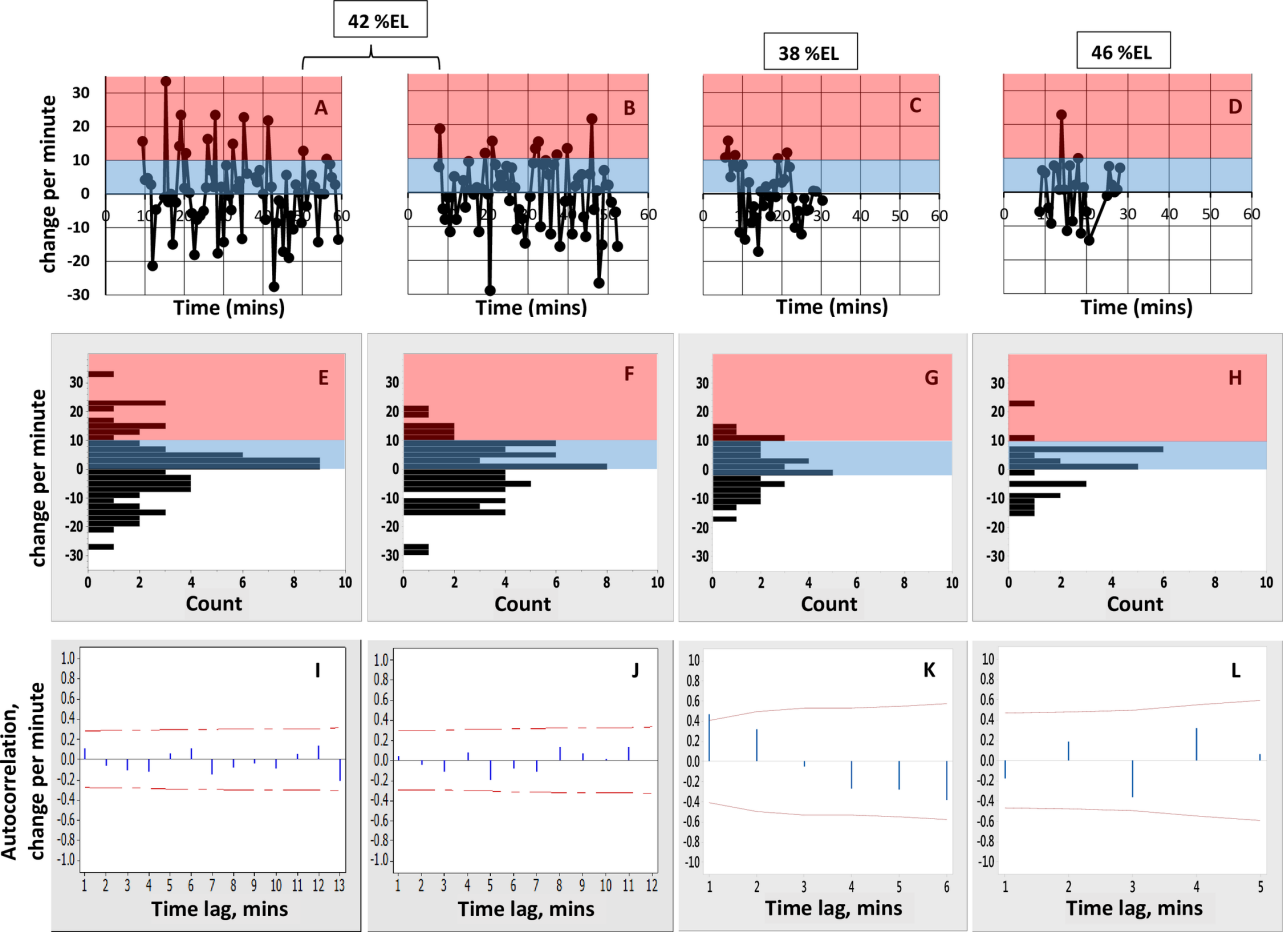
Statistics for per-minute changes in the experimental time series. (A-D) Change per minute for the experimental time series at stripe-center (A, B; data of Fig 1B and 1C, respectively); the anterior stripe edge (C; data of Fig 1D); and the posterior stripe edge (D; data of Fig 1E). Change per minute is calculated as the difference in number of nascent transcripts divided by the difference in time for each pair of successive data points. This is the net change (*number of initiating transcripts*–*number of terminating transcripts* per minute), i.e. intervals with more initiation than termination have positive change. Pink indicates high initiation intervals (gains of 10 or more nascent transcripts per minute); blue indicates low to mid initiation intervals (below 10 additions per minute). The threshold of 10 is for illustrative purposes, it is not used in the statistical analysis of the experimental data or model results. (E-H) Corresponding histograms of the per-minute changes (E ↔ A; F ↔ B; G ↔ C; H ↔ D), labeled at lower class limits. (E, F) At stripe-center, the distribution of change rates is broad, ranging from a maximum net addition of 33.5 transcripts in a minute (A, minute 15) to a maximum net loss of 29 transcripts in a minute (B, minute 20). While low net-change minutes (near zero addition) are most common, mid- to high-change minutes are also well populated. Approximately 55% of per-minute changes are positive. (G, H) At the stripe edges, with higher concentrations of the Gt and Kr repressors, the distribution of rates becomes narrower: to the anterior (G), at 38%EL, the histogram is well-filled for low to medium rates, but shows less frequent high intensity intervals than at stripe-center; to the posterior (H), at 46%EL, while high-intensity minutes can still be accessed (e.g. D, minute 14), per-minute change is predominantly low to mid, and the distribution is more uneven than at 38%EL, with low to zero frequency for many of the rates. (I-J) Autocorrelation plots for the corresponding per-minute changes (I ↔ A; J ↔ B; K ↔ C; L ↔ D). The per-minute changes show no significant autocorrelation at stripe-center or the posterior edge (I, J, L; red lines are 5% significance limits, zero ± t*SE, see text), and autocorrelation at 1 minute lag for the data at the anterior edge of the stripe (K).

The change in the number of nascent transcripts from minute to minute provides information on transcription rates at the resolution of the data. Fig 3A–3D show the change per minute for the data in Fig 1B–1E. These show large minute-to-minute changes in number of nascent transcripts. For example, Fig 3A shows minutes with net additions of over 20 transcripts, indicating a relatively high initiation rate, followed the next minute by net loss of transcripts, indicating a lower initiation rate. These per-minute changes shows no significant autocorrelation at any time lag for the data at stripe-center or posterior edge (Fig 3I, 3J and 3L). High and low addition minutes are largely mixed together, without a persistence in the per-minute rate. I.e., nascent transcript peaks consist of sub-intervals with highly varying rates of change (see Fig 1B–1E). The zero autocorrelation (or very short, Fig 3K shows a 1 minute lag) in these per-minute changes indicates that there is no basis for associating a particular transcriptional initiation rate with an extended interval, such as the 4–10 minute duration of a nascent transcript burst peak: these peaks cannot be used as evidence for multiple ON-states.

### Parameter estimation

Model parameters (rate constants *k*, Fig 2) can be estimated from the experimental data in a sequential manner, first for Bcd, then for Hb, then for Gt and Kr. These steps are outlined below and correspond to the green circles in Fig 2. The spatial patterns of the TFs (S1 Fig) and experimental interventions (e.g. [8,25]) indicate that *eve* is activated throughout the anterior by Bcd and Hb and that stripe 2 forms where there is a relative lack of repression, at the trough between Gt to the anterior and Kr to the posterior. Steps 1–6 below are set by stripe-center data, assuming that Gt and Kr do not bind appreciably here (if they did, the Bcd and Hb binding rates would scale up proportionally). Parameter estimation starts with rates involving only Bcd (steps 1 and 2), using statistics from the experimental time series and results for the mutagenized hb-3 BS (ibids.). Rates involving Hb (steps 3 and 4) are set to match expression for the full reporter construct (e.g. Fig 1B and 1C). Gt and Kr rates (step 7) are set to match expression at the anterior and posterior stripe edges, respectively.

**Fig 4:**
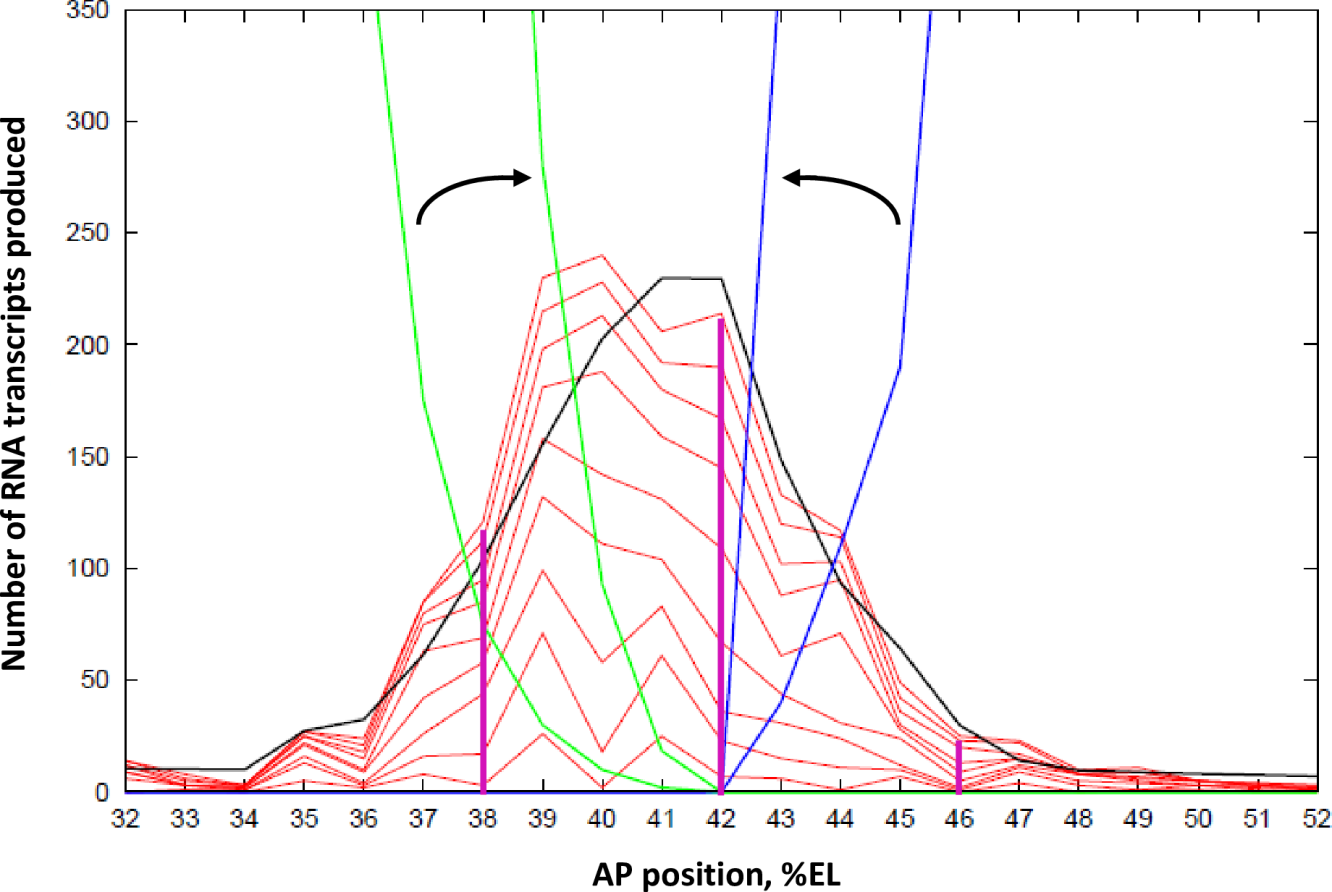
Spatial patterning of the *eve2* mRNA stripe: Number of mRNA molecules produced vs. AP position (in %EL). Black: deterministic (no-noise) solution of the MSE model (Fig 2) for *eve* mRNA produced by 45 minutes of transcription. This corresponds to the accumulated mRNA from Fig 3D of [4], found by integration of the nascent transcript signal over time. Activation is by Bcd and Hb, which both have high expression anterior of 49%EL (S1 Fig). The Gt (green) and Kr (blue) repressors are at zero concentration at 42%EL. The Kr gradient to the posterior and the Gt gradient to the anterior repress *eve*2 expression increasingly with distance from the stripe-center. Ten-fold increase of Kr and Gt over nc14 (outer to inner green and blue lines, arrows) produces the experimentally observed sharpening of the stripe. Moderate *eve*2 expression extends more anterior (to ~37%EL) than posterior (to ~45%EL), matching experimental data, due to the shallower Gt than Kr gradient. The red lines are 5-minute separated intervals of a stochastic simulation of the model, minutes 5 to 45 shown.

1. *(Fig 2, green circle 1)* Bcd activation alone, MSE binding: Roughly 55% of the time intervals in Fig 3A and 3B show an increase in nascent transcripts. Since Bcd is necessary for transcriptional initiation, this can be identified with 55% fractional occupancy of the E[1x00] state (stochastically, the proportion of time spent in this state). Kinetically, this represents an equilibrium between Bcd BS binding and unbinding rates. For the Bcd unbinding rate constant, *k*_UNBIND-B_, set by step #5 below, 55% fractional occupancy corresponds to a Bcd binding rate constant, *k*_BIND-B_, of 2.6e6 M^-1^ s^-1^ (for the Bcd concentration [B] in S1 Fig—*k*_BIND-B_*[B] is constant for given fractional occupancy and *k*_UNBIND-B_; the concentration scale in S1 Fig was determined as in [29], [B] is independently corroborated by the levels reported in [32]).
2. *(Fig 2, green circle 2)* Bcd activation alone, LOW initiation rate: Fig 3D of [4] reported mean production of 230 *eve*2 transcripts with the intact *eve2* DNA regulatory element (Bcd plus Hb co-activation) at stripe-center over nc14 (~45 mins. of transcription). Mutagenesis of the hb-3 BS reduces *eve2* expression [8,25]. If such Bcd-only activation were half-normal, this would correspond to, on average, 115 transcripts over nc14. This sets *k*_1000_, the rate constant for transcriptional initiation in the E[1000] state, to 0.09 s^-1^ (or 5.4/min). (If the hb-3 effect was not exactly half, *k*_1000_ would scale accordingly.)
3. *(Fig 2, green circle 3)* Bcd plus Hb co-activation, HIGH initiation rate: The maximum observed per-minute increase is +33.5 molecules in one minute (at minute 15 in Fig 3A). Using this for the maximum initiation rate when both Bcd and Hb are bound (the E[1100] state) sets *k*_1100_ to 0.56 s^-1^.
4. *(Fig 2, green circle 4)* Bcd plus Hb activation, MSE binding: With this *k*_1100_, Hb binding, *k*_BIND-H_, needs to be 1.8e5 M^-1^ s^-1^ (for the Hb concentration in S1 Fig) to produce the observed average of 230 transcripts over nc14 (115 in addition to the 115 in step 2).
5. *(Fig 2, green circles 5)* Unbinding rates from the BSs (*k*_UNBIND-B_, *k*_UNBIND-H_, *k*_UNBIND-K_, *k*_UNBIND-G_) are assumed to be the same for all TFs (if they were unequal the binding rates would scale accordingly). The unbinding rate constant value (= 0.033 s^-1^, or 2/min) is constrained by matching the stripe-center observations of both **a**) autocorrelation for 1–3 minute lags in the number of nascent transcripts (S2 Fig) and **b**) no autocorrelation (at any time lag) in the per-minute change in number of nascent transcripts (Fig 3I and 3J). A particular fractional occupancy (step 1, above) corresponds to a particular ratio of *k*_BIND_ to *k*_UNBIND_. But faster absolute values of these *k*’s will smooth out fluctuations and reduce autocorrelation on the minute timescale, while slower *k*’s give more persistence of states and increase autocorrelation. The *k*’s need to be slow enough to generate condition **a** (i.e. to generate bursting in nascent transcript number), but not so slow as to violate condition **b**.
6. (*Fig 2, green circle 6*) Transcriptional termination, producing a completed, free mRNA molecule, is modeled as a first-order process. The rate constant, *k*_T_ = 3.97e-3 s^-1^, corresponds to a mean transit time of the coding region of 4.2 minutes [4], based on a mean velocity for transcriptional elongation from [13]. The lack of autocorrelation in the per-minute changes (Fig 3I and 3J) supports that initiation is independent of termination: there are no ‘un-bursts’ of termination correlated to earlier ‘bursts’ of initiation (at any time lag), perhaps due to variability in elongation times.
7. (*Fig 2, green circles 7 and 8*) Kr and Gt binding constants are set to match mRNA produced to the experimental values at all AP positions from 35 to 47%EL and times of 15, 30 and 55 minutes into nc14 (Fig 3D of [4]). A ten-fold increase in Kr and Gt concentrations over nc14 (S1 Fig), modeled as a first order translation from fixed mRNA concentration gradients, generates the observed sharpening of stripe 2 over nc14 (Fig 4). Kr binding constant, *k*_BIND-K_ = 9.9e6 M^-1^ s^-1^; Gt binding rate constant, *k*_BIND-G_ = 1.98e7 M^-1^ s^-1^.

## Results and discussion

### Spatial patterning

**Fig 5:**
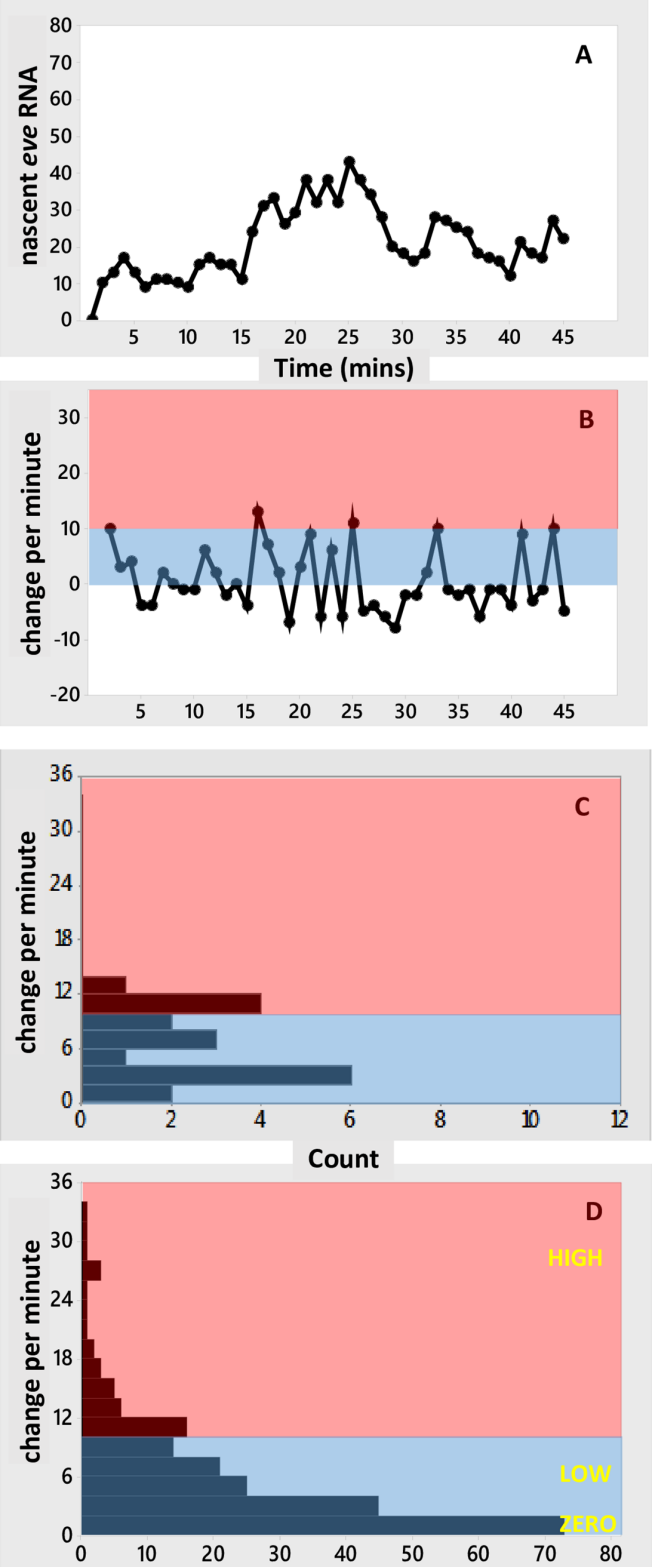
Time series at stripe-center. Simulated time series with activation by Bcd (E[1000] state, LOW initiation rate) and Bcd+Hb (E[1100] state, HIGH initiation rate), and no repression (at this position). Same simulation as Fig 4, sampled at the magenta line at 42%EL. (A) Number of nascent transcripts vs. time, generating the bursting seen in the experimental time series (Fig 1B and 1C). (B) Per-minute change vs. time, showing the mixing of high and low initiation intervals. Pink, blue as in Fig 3. (C) Histogram of the per-minute changes. (D) Histogram of per-minute change pooled from 10 replicate simulations (S4 Fig). The steady decrease in frequency with increasing rate in (D), with well-populated low to mid rates (blue), follows the trend in the data (Fig 3E and 3F). HIGH, LOW and ZERO labels correspond to *k*_1100_ = 33.5/min, *k*_1000_ = 5.4/min, *k*_0000_ = 0/min, respectively.

**Fig 6:**
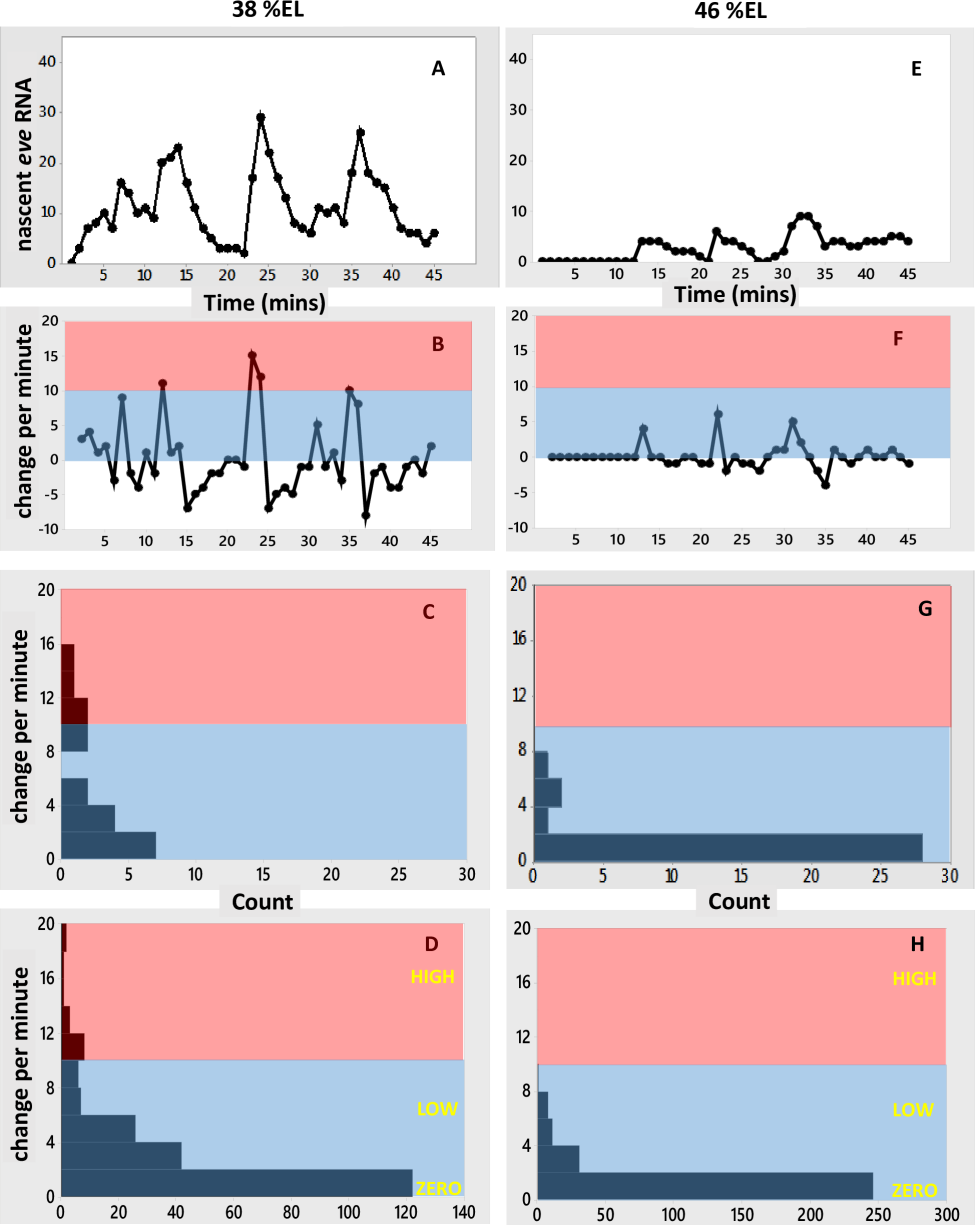
Time series at stripe-edge positions. (A-C) anterior edge of the stripe, at 38%EL, same simulation as Figs 4 and 5, corresponding to Figs 1D and 3C, 3G and 3K data. (A) Number of nascent transcripts vs. time. (B) Per-minute change vs. time. (C) Histogram of per-minute changes. (D) Histogram of per-minute change pooled from 10 replicates of the simulation (S5A–S5C Fig). (E-G) same simulation at the posterior edge of the stripe, 46%EL, corresponding to Figs 1E and 3D, 3H and 3L data. (E) Nascent transcripts vs. time. (F) Per-minute change vs. time. (G) Histogram of per-minute changes. (H) Histogram of per-minute change pooled from 10 replicates (S5D–S5F Fig). In general, repression decreases the occurrence of high initiation intervals and increases the frequency of low initiation intervals, compare (D) and (H) to the stripe-center (non-repressed) expression in Fig 5D. The moderate expression (under moderate Gt repression) at 38%EL (D) has a wider range of per-minute changes than the low expression (under stronger Kr repression) at 46%EL (H). The HIGH Bcd+Hb rate (*k*_1100_) is seen under moderate repression (D), it is not observed under strong repression (H). HIGH, LOW and ZERO labels as in Fig 5. Pink, blue as in Fig 3.

With the above parameters and the TF spatial patterns in S1 Fig, deterministic solution of the MSE model (Fig 2) generates a spatial peak of *eve2* mRNA product (Fig 4, black). The deterministic solution is matched to the experimental profiles for average accumulated mRNA per nucleus (Fig 3D of [4]) at 5, 20, and 45 minutes of transcription. Increase of Gt (Fig 4, green) and Kr (Fig 4, blue) concentrations over nc14 transforms an initial broad *eve2* stripe into the later sharp stripe. The red lines in Fig 4 are at 5-minute intervals of a stochastic solution of the model. These show the lower stripe-edge production compared to stripe-center, corresponding to stripe narrowing, as well as the stochastic, uneven production of mRNA produced at each location (40%EL, for example, is quite uneven in this simulation). Variability in outcomes is shown for 10 replicates of the stochastic simulation in S3 Fig. At each AP position, time series can be extracted for comparison to live expression data (Fig 1B–1E; Fig 3). Magenta lines indicate positions at which time series are analyzed in subsequent sections: at stripe-center, 42%EL (Fig 5); and at the stripe edges, to the anterior (38%EL) and to the posterior (46%EL), Fig 6.

### Time series

#### Bcd and Hb activation, stripe-center

At the *eve2* peak, MSE regulation has minimal repression compared to activation. Representing this in the model as zero repressor concentration at 42%EL (Fig 4), transcriptional initiation at stripe-center depends only on activation by Bcd and Hb binding (state of E[xx00], 3^rd^ and 4^th^ positions always 0). Stripe-center time series from stochastic simulations (Fig 5A; S4A Fig for replicates) generate the nascent transcript bursting seen in the data (Fig 1B and 1C), including 1 to 3 minute autocorrelation. Per-minute changes (Fig 5B; replicates in S4B Fig) show high intensity initiation minutes (state E[1100], HIGH rate constant *k*_1100_) interspersed with low intensity minutes (state E[1000] with LOW rate constant *k*_1000_, or very transient E[1100]). As with the experimental time series, no autocorrelation is observed in the per-minute changes. The per-minute addition of transcripts shows a relatively even distribution from lower to higher values (Fig 5C; replicates in S4C Fig). Pooling the S4C Fig histograms indicates a distribution of rates that has high probability for low initiation minutes and tails off to high intensity addition minutes (Fig 5D), similar to the experimental distribution (Fig 3E and 3F).

#### The effect of repression: Stripe-edge expression

Transcriptional repression increases with distance from the *eve2* stripe-center, due to the Gt gradient to the anterior (increasing occupation of the E[xxx1] state) and the Kr gradient to the posterior (occupation of the E[xx1x] state). These gradients shape the expression stripe spatially (Fig 4). Fig 6 shows time series at the 38%EL (Fig 6A) and 46%EL (Fig 6E) positions (extracted from the Fig 4 simulation; time series of 10 replicates shown in S5 Fig). 4%EL to the anterior of stripe-center, at 38%EL, Gt repression is moderate (relative to Bcd and Hb activation), and *eve2* expression reaches roughly half-maximal levels (matched to data from [4], see Fig 4). The time series (Fig 6A) shows bursting peaks, like the experimental data (Fig 1D), with low to moderate levels of per-minute change (Fig 6B–6D). 4%EL to the posterior of stripe-center, at 46%EL, Kr repression is high (relative to Bcd and Hb activation) and *eve2* expression is quite low (see Fig 4). Several small amplitude bursts are seen in the time series (Fig 6E and 6F), and only low per-minute rates are seen (Fig 6G and 6H). Anterior positions, with moderate Gt repression (Fig 6D), show a wider range of per-minute changes than posterior positions, under stronger Kr repression (Fig 6H).

This relative difference between anterior and posterior stripe edges also appears in the data, with a more ‘filled in’ histogram consistently accessing moderate per-minute changes at 38%EL (Fig 3G) compared to a more sporadic, lower per-minute histogram at 46%EL (Fig 3H). In the experimental data, the early shut-off of stripe-edge transcription (Fig 1D and 1E), with moderate transcription levels while on, may reflect effects from global positional shifts in repressor patterns (e.g. see [33]).

### Loss of co-activation: reduced expression with mutation of the Hb BS

**Fig 7:**
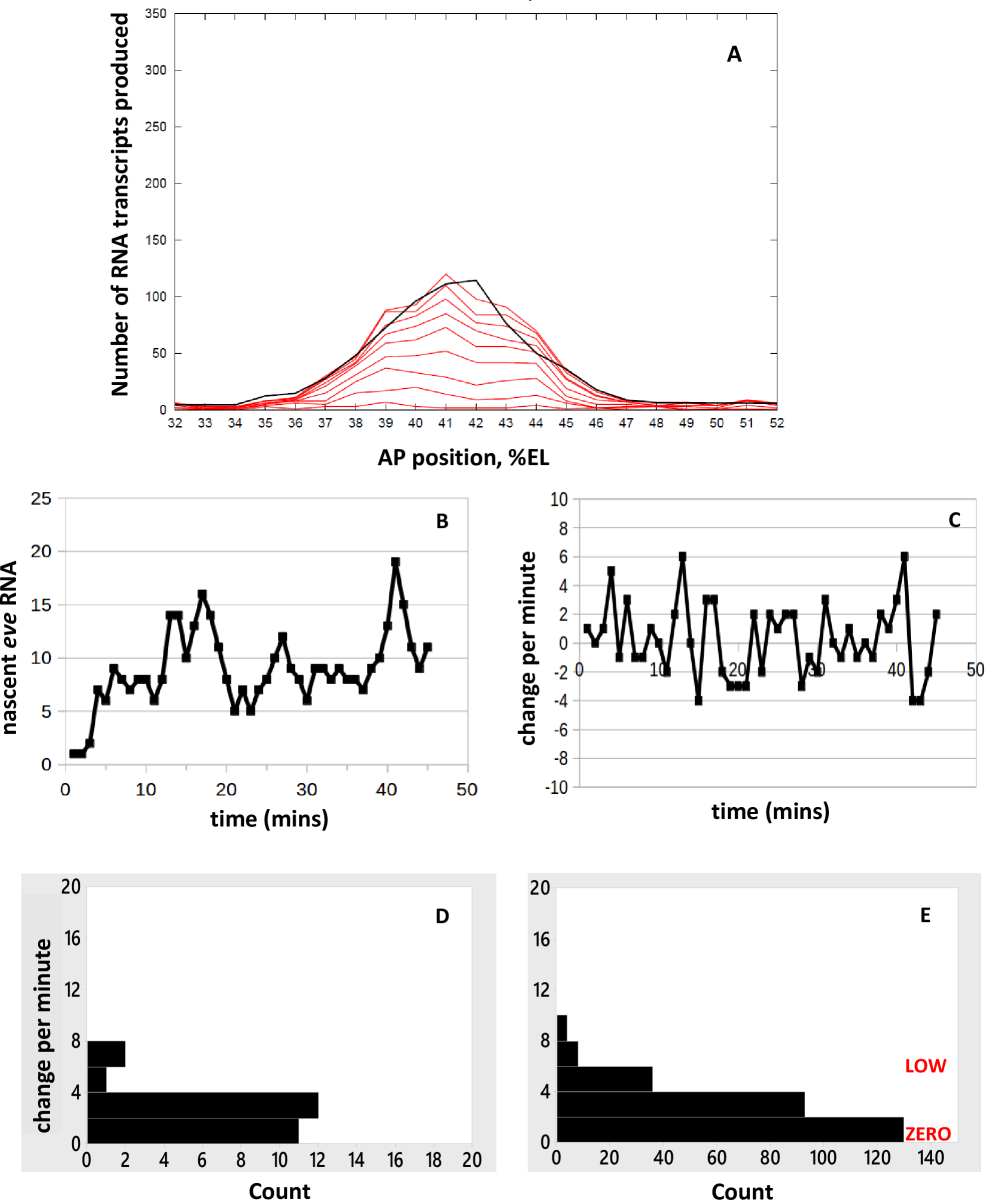
Prediction of *eve2* expression without Hb co-activation. (A) Spatial pattern of RNA production. Black line, deterministic solution at 45 minutes; red lines, 5-minute separated intervals of a stochastic simulation, 5 to 45 minutes shown. 10 replicate simulations shown in S6 Fig. (B-D) Time series at stripe-center (42%EL). (B) Number of nascent transcripts vs. time. (C) Per-minute change in nascent transcripts. (D) Histogram of per-minute change. (E) Histogram of per-minute change pooled from 10 replicate simulations (S6D Fig). Bursting is still expected for constructs lacking Hb co-activation, but without the HIGH intensity intervals seen for the intact MSE. LOW, ZERO as in Fig 5. Pink, blue as in Fig 3.

Fig 7 shows model results without Hb co-activation, in which binding of Hb (*k*_BIND-H_) is set to zero to simulate mutagenesis of the Hb BS as in [8,25]. In this case, all transcriptional initiation is from the E[1000] state, at the LOW rate (*k*_1000_ = 0.09 s^-1^). Stripe-center expression (Fig 7A, black line, deterministic solution) is half that for E[1000] plus E[1100] activation (Fig 4), reflecting the experimental reduction of expression when the Hb BS is mutagenized. The 5-minute separated intervals of the stochastic solution (red lines, Fig 7A) indicate the dynamics which could be expected with Hb co-activation blocked (10 replicates shown in S6 Fig). In the time series (Fig 7B), bursting is still observed, though the number of nascent transcripts is lower than with full Bcd and Hb activation (Fig 5). The per-minute changes are all of ZERO and LOW intensity (Fig 7C–7E). Even with a single initiation rate constant (*k*_1000_) in these simulations, a distribution of rates is observed, due to the noisy occupation time of the E[1000] state as well as fluctuations in initiation events while in E[1000].

The distribution of per-minute changes for loss of co-activation (Fig 7E) contrasts with that seen for increased repression at the stripe edges (Fig 6). Stripe-center E[1000]-activated expression (Fig 7A) produces the same amount of RNA product (~half-maximal) as co-activated/Gt-repressed expression at 38%EL (Fig 4). Even with Gt repression, we predict that co-activated HIGH intensity intervals should still be observed at 38%EL (Fig 6D; compare experimental histogram, Fig 3G), while expression from a construct without Hb co-activation should show only ZERO and LOW intensity intervals (Fig 7E). Co-activated expression at the posterior edge (Fig 6H) shows a similar ZERO and LOW-only distribution of per-minute changes as expression at stripe-center without Hb co-activation (Fig 7E). However, the strong Kr repression at 46%EL (Fig 6H) makes the ZERO intervals more common than for unrepressed expression lacking Hb co-activation (Fig 7E); at the stripe-center, the E[1000] LOW state is sufficiently occupied to produce half-normal RNA product (Fig 7A).

### Test of one transcription ON-state (simple ON-OFF model) for full expression

Noisy bursting in gene expression has been studied in terms of a simple ON-OFF transcription model, in which a single ON transcription state, with a characteristic rate constant, is occupied stochastically (see review in [34], and recent work in [35] on spatial variability of the *eve2* stripe border positions). The current model allows us to test whether a simple ON-OFF mechanism could generate the characteristics of the experimental time series, or whether the two-ON-state E[1000], E[1100] MSE mechanism produces a closer fit. If the two-ON-state mechanism offers a better fit, this indicates that features of the cis-regulatory structure can be extracted from noisy time series; if, on the other hand, the one-ON-state and two-ON-state fits to the data cannot be distinguished from one another, this suggests time series cannot be used to select a unique regulatory mechanism, for the rate parameters indicated by the *eve2* data.

**Fig 8:**
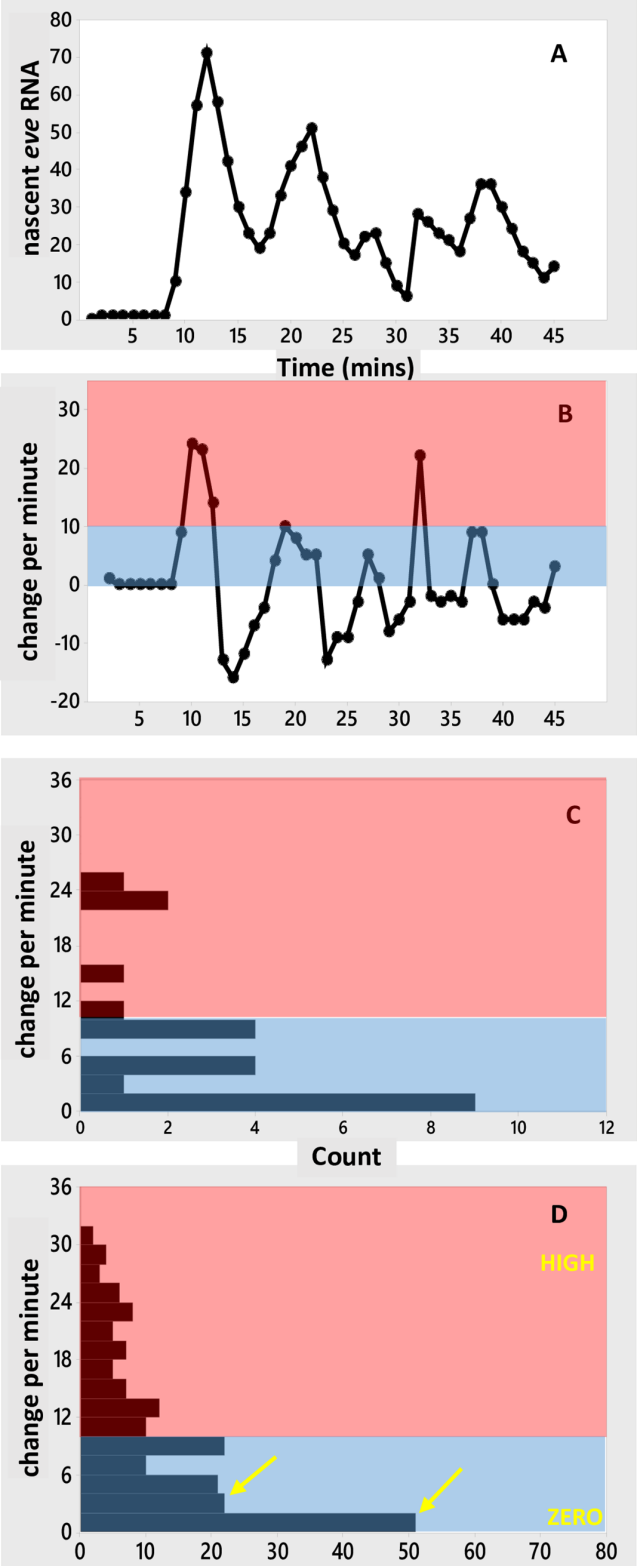
Test of a simple ON-OFF model, with a single ON transcription state. Simulated time series at stripe-center (42%EL) with activation only from the E[1000] state, but producing normal levels of RNA. Fig 7 models LOW rate E[1000] transcription, with reduced RNA levels, for loss of the HIGH rate E[1100] pathway; Fig 8 tests the capacity for a single state, E[1000], to model the experimental time series for the intact *eve2* reporter with ON transcription only at the HIGH rate (highest experimentally observed additions per minute, Fig 3A). (A) Number of nascent transcripts vs. time. (B) Per-minute change vs. time. (C) Histogram of per-minute changes. (D) Histogram of per-minute change pooled from 10 such simulations (S7 Fig), showing a sharp drop in count from the near-zero bottom bar (0–1 additions per minute) to the next bar (2–3 additions per minute; see arrows, compare Fig 5D), and a low but broad distribution up to the maximum observed rate. This distribution is due to noise in occupancy of the E[1000] state and transcriptional initiation, not due to multiple ON-state transcription. HIGH, ZERO as in Fig 5. Pink, blue as in Fig 3.

To simulate the capacity of a one-ON-state mechanism to generate characteristics of the experimental time series, we removed Hb co-regulation by setting *k*_BIND-H_ to zero, leaving Bcd as the sole activator (consistent with its necessity in *eve2* transcription [8]). Transcriptional initiation is solely from the E[1000] state, but in this case RNA output needs to be full-scale, to produce the observed mean 230 transcripts in nc14. The *k*_1000_ rate constant was set to the maximum observed HIGH rate in the data (0.56 s^-1^). These simulations are therefore unlike the E[1000] simulations of Fig 7, which simulated the effect of mutagenesis of the Hb BS and the resulting reduction in RNA output from transcription at the LOW rate. To match full RNA output with a HIGH *k*_1000_, *k*_BIND-B_ was decreased to 18% of the two-ON-state MSE model (to 4.75e5 M^-1^ s^-1^). Time series of nascent transcript numbers from these simulations show low and high ‘bursting’ peaks (Fig 8A; S7 Fig for 10 replicates), with significant autocorrelation at lags of 1 to 3 minutes, similar to the experimental data (Fig 1B and 1C; S2 Fig). These results indicate that multi-minute bursts are not indicative of a multiple ON-state mechanism, since they can be generated with the simple ON-OFF model.

The per-minute increases in transcript (Fig 8B) show a broad range of values (Fig 8C and 8D). In this case, however, there is no contribution from mixing 2 ON rates (LOW and HIGH) as in the MSE model (Fig 5). Since ON transcription is at a single HIGH level in the simple ON-OFF model, the distribution across rates in Fig 8D is due to noise in the occupancy of the activator BS (value of the random variable x in E[x000]) and variability of initiation events while in E[1000].

Significant autocorrelation in per-minute changes is found in 5 out of 10 simulations (S7 Fig). This is unlike the experimental data (Fig 3I and 3J) or the two-ON-state simulations, which show no significant autocorrelation. In addition, the histogram of per-minute changes shows a sharp difference between the lowest initiation minutes (lowest bar in Fig 8D, 0–1 transcripts added per minute) and higher rates (all higher bars in Fig 8D, 2 or more transcripts added per minute), which is not seen with the experimental data (Fig 3E and 3F) or the two-ON-state mechanism (Fig 5D). The two-ON-state mechanism (Fig 5D) has a narrower distribution of rates: very high addition minutes are less likely and there is a smoother transition from the lowest intensity minutes to the higher rates than with the simple ON-OFF model (Fig 8D). A χ^2^ test of independence (which can be applied across the 8 bars with counts > 5) supports the visual comparison of the histograms, with *p* = 0.037 indicating the two-ON-state and one-ON-state mechanisms have different distributions of rates. The strongest differences (contributors to χ^2^) are at 2–3 additions per minute (2^nd^ bar), reflecting the larger drop between the 1^st^ and 2^nd^ bar in the one-ON-state vs. two-ON-state mechanisms, and at 8–9 and 12–13 additions per minute (5^th^ bar and 7^th^ bar), reflecting the wider distribution with one-ON-state than two-ON-states.

In the one-ON-state mechanism, there are only ZERO or HIGH transcription rates, and occupancy of the ON rate (17% in E[1000] in Fig 8) must be lower than in the two-ON-state mechanism (54% total for E[1000] and E[1100] in Fig 5) to produce the same mean amount of RNA in 45 minutes. I.e., for the same target production, the one-ON-state mechanism has more OFF time (83%) and the ON intervals are higher intensity than with the two-ON-state mechanism (which has 46% OFF time). The one-ON-state model produces a more dichotomous HIGH-OFF distribution of rates, while the two-ON-state model is ON more consistently, with LOW intervals (43.2% of the time in Fig 5) maintaining transcription between HIGH spikes (10.4% of the time in Fig 5).

In Fig 7E, the one-ON-state ZERO-LOW mechanism shows a smoother transition between ZERO and positive intervals than the one-ON-state ZERO-HIGH mechanism (Fig 8D). The ZERO-LOW model is a simulation of loss of Hb co-activation and reduced *eve2* production, with E[1000] occupancy the same as E[1x00] occupancy in the two-ON-state mechanism (54% ON time). The ZERO-HIGH model is a test of a single HIGH rate to produce normal levels of *eve2* RNA, and this HIGH rate requires a fractional occupancy of 17% for the activator, with 83% of the time OFF from transcription, strongly populating the lowest bar of the per-minute change histogram.

The experimental time series indicate a more even distribution of zero to low initiation rates (blue, Fig 3E and 3F) than is seen with the one-ON-state model (Fig 8D). This even distribution is more indicative of a contribution from the LOW rate in the two-ON-state model (Fig 5D). In addition, for all parameters the same except for *k*_BIND-H_ (set to zero for one-ON-state) and *k*_BIND-B_ (reduced for one-ON-state), the one-ON-state model violates the lack of autocorrelation seen in the experimental per-minute changes.

### Variation of parameters

The parameters used in the model are estimated from a number of experimental data features (*Parameter estimation*, Methods). To test the generality of the characteristics seen in the model time series, we ran series of computations varying parameters consistent with experimental error limits. Activation parameters in the model are set by four experimental observations: **a**) the fraction of minutes with net addition of transcripts, setting the fractional occupancy of the activator BSs (step 1, *Parameter estimation*, Methods); **b**) the highest observed rate of nascent transcript increase, setting the HIGH initiation rate (step 3); **c**) the mean *eve2* RNA produced in nc14, setting the LOW initiation rate (step 2) and the occupancy of E[1100] (step 4); and **d**) autocorrelation, setting the absolute rates of TF binding and unbinding (step 5). Noise can be considered primarily in terms of the fractional occupancy of the TF BSs (**a**) and the rates of BS binding and unbinding (**d**), with steadier output for higher fractional occupancy (ON time) and faster binding and unbinding (temporal averaging). Variations in maximum initiation rate (**b**) and RNA output (**c**) ultimately affect **a** and **d**, with variations in **b** while holding **c** constant, and vice-versa, requiring adjustments in **a** or **d**.

For example, the possibility that mean RNA output is higher than the experimental sample mean in [4] can be simulated by relaxing the constraint on RNA output (**c**) for steps 2 and 4. While keeping the constraints on total activator BS occupancy (**a**, Step 1) and maximum initiation rate (**b**, step 3), increasing the LOW initiation rate increases the mean half-maximal output (i.e. more than 115 transcripts generated via E[1000], step 2) and increasing Hb binding increases the mean maximal output (i.e. more than 115 transcripts generated via E[1100], step 4). The unbinding rate then needs to be increased to maintain the autocorrelation constraint (**d**). This narrows the distribution of initiation rates and decreases the observed differences between the one-ON-state (e.g. Fig 8D) and two-ON-state (e.g. Fig 5D) histograms. For instance, in a set of simulations with mean RNA output raised to 245, similarity between the histograms increased and gave a χ^2^ test with *p*>0.05. Similar results were seen for holding unbinding (**d**) fixed while increasing total BS occupancy (**a**).

Likewise, the possibility that the HIGH rate is lower than observed in [4] can be simulated by relaxing constraint **b** for Step 3, and retaining constraints **a** (step 1) and **c** (steps 2 and 4). This requires faster binding and unbinding to maintain the autocorrelation constraint (**d**). For instance, a set of simulations with the HIGH rate reduced to 22/min (a value observed multiple times, Fig 3A and 3B) visually showed narrower and more similar histograms between one-ON-state and two-ON-state (though χ^2^ had *p*<0.05, largely due to differences in width, at the 5^th^ bar). As above, holding unbinding (**d**) fixed while increasing BS occupancy (**a**) would be expected to similarly reduce histogram width. Conversely, cases with lower mean RNA or a higher HIGH rate than the experimental values in [4] could correspond to lower fractional occupancy (**a**) or slower binding and unbinding (**d**), which would produce output that had more OFF time or was less time-averaged than in Figs 5–8, aiding the distinction between one-ON-state and two-ON-state distributions.

The estimated values for the model parameters (Methods) indicate that characteristics from the two-ON-state mechanism can be detected in the experimental time series for *eve2*. However, the one-ON-state/two-ON-state distinction could become obscured if TF binding was faster than indicated. In such a case, and without a corresponding increase in TF unbinding, fractional occupancy (ON time) would increase, producing either more RNA or needing a lower maximum initiation rate. If the TF unbinding rate was matched to the binding increase, RNA and initiation rates could be unaffected, but temporal averaging would increase. It is also a possibility that the fit of the one-ON-state mechanism to the experimental time series could be improved if it ran at faster TF binding/unbinding kinetics than the two-ON-state mechanism. In general, it may be difficult to identify multiple ON-state transcription from time series in developmental systems with TF binding much faster than appears to be the case for *eve2*. But for systems with kinetics on the order of or slower than estimated here, the current modeling approach, of comparing one-state and two-state output to experimental time series, could likely distinguish multiple ON-state characteristics of the transcriptional mechanism.

### Predictions for two active gene copies

Bothma et al. [4] speculated that having two actively transcribing copies of a gene could contribute to the appearance of multiple ON-states. Their data was collected on one transcribing spot per nucleus, and all of the above modeling was likewise done with one transcribing center per nucleus. However, we can extend the model to see what features might arise from dual-copy transcription per nucleus. For these simulations, each nucleus had two independent copies of the model (Fig 2), each contributing (on average) half of the total transcription (i.e. the LOW and HIGH initiation rates were halved from the single-copy simulations). In general, 2-copy time series still show bursting in the total nascent transcript per nucleus, whether one-ON-state or two-ON-state. Histograms of change per-minute show that 2-copy transcription (S8C Fig, two-ON-states; S8D Fig, one-ON-state) narrows the distribution of initiation rates compared to 1-copy transcription (S8A Fig, two-ON-states; S8B Fig, one-ON-state), in particular filling the low initiation intervals for the one-ON-state mechanism (S8 Fig, arrows). For dual copies there are more total initiation states than for one copy. For example, the simple ON-OFF, ZERO-HIGH mechanism has ZERO-ZERO, ZERO-HIGH, and HIGH-HIGH states with 2 copies per nucleus (cf. discussion in [4] of multiple ON-states from multiple copies), and the ZERO-LOW-HIGH mechanism has 6 such combined states. This decreases the probability of achieving the highest rate (both copies initiating at HIGH-HIGH) and increases the chance for the nucleus to be in a lower initiation state, as reflected in the narrowing of the histograms from 1-copy to 2-copy simulations. From this, we predict that measurements of summed signal from 2 actively transcribing gene copies per nucleus (e.g. nuclear dots) should be more consistent and show less extreme events than measurements of a single transcribing center, which may make it more difficult to distinguish multistate from simple ON-OFF transcription.

## Conclusions

We have developed a mathematical model of *eve2* gene expression driven by the four anterior TFs controlling transcription in the stripe 2 region through the MSE cis-regulatory element (Fig 2). All parameters in the model can be estimated and constrained by features in the experimental time series [4] and earlier experimental results on the TF binding sites in the MSE [8,25].

Stochastic simulations generate ensembles representing the spatial and temporal variability to be expected for *eve2* expression subject to particular types of regulation. From these, we can identify different noise characteristics with particular aspects of *eve2* activation and repression. TF BS studies indicate that Bcd is a necessary activator of *eve2*, and Hb is a co-activator which enhances expression [8,25]. Stochastic simulations of this dual activation, with a LOW transcriptional initiation rate in the Bcd-only E[1000] state of the MSE and a HIGH initiation rate in the Bcd+Hb bound E[1100] state recreate the noisy bursting observed in the experimental time series and show a smoothly varying distribution of initiation rates between ZERO and HIGH (Fig 5).

Repression at the stripe edges limits the extent of *eve2* expression. At the anterior edge, there is moderate *eve2* expression, with Gt repression and relatively higher Bcd and Hb activator concentrations. At the posterior edge, *eve2* has low expression, with Kr repression and relatively lower Bcd and Hb concentrations. We predict that nuclei at the anterior edge should show diminished ON rates compared to stripe-center, but that intervals of HIGH initiation should still be observed. To the posterior, we predict even fewer ON intervals, and that HIGH initiation rates would be much less common (Fig 6), mirroring the observations in the experimental time series (Fig 3G and 3H).

Mutagenesis of the Hb BS leads to reduced *eve2* expression [8,25]. While this may produce comparable levels of *eve2* expression to moderate anterior-edge expression for the intact MSE, we predict that loss of Hb co-activation should show different time series features than anterior Gt repression: for loss of Hb co-activation, HIGH initiation rate intervals should not be observed (Fig 7).

The stochastic model allows us to test whether the experimental time series indicate multiple ON transcription states, with distinct rates, or whether the data is consistent with a simple ON-OFF model of transcription, with a single ON-state. We tested, first, whether time series generated by one-ON-state and two-ON-state mechanisms significantly differ, and second, if so, which provides a closer fit to the experimental time series. In other words, can the combination of a low Bcd-only activation and higher Bcd+Hb co-activation be detected, and can the experimental time series be used to make inferences about the regulatory mechanism?

Simulations indicate that both the two-ON-state MSE mechanism and a one-ON-state mechanism (without Hb co-activation) can generate the multi-minute bursts in number of nascent transcripts seen in the experimental time series. The regulatory mechanism cannot be inferred from the data at this temporal resolution. However, at the level of per-minute changes the mechanisms show differences, with the one-ON-state mechanism showing a strong drop in prevalence from the zero (or extremely low) to higher initiation intervals (Fig 8), and the two-ON-state mechanism (Fig 5) and experimental data (Fig 3) showing a more continuous distribution of rates. For the parameters estimated from the data (Methods), modeling indicates that the one-ON-state and two-ON-state mechanisms can be distinguished and that the two-ON-state distribution of rates gives a closer fit to the *eve2* data; i.e. that features of the Bcd-only and Bcd+Hb dependent transcription rates can be detected in the experimental time series, and that these features, particularly low per-minute changes, are missed by a one-ON-state model.

Fluctuations are damped and rate distributions narrow as either TF BS fractional occupancy or the rate of TF binding and unbinding increase. This would be predicted to make the difference between the one-ON-state and two-ON-state mechanisms less distinct. Our approach, of looking for multistate transcription by evaluating the output of potential mechanisms against experimental time series, should be applicable across developmental systems as long as binding kinetics are sufficiently slow. In addition, we predict that differences in the one-ON-state and two-ON-state mechanisms should be easier to detect from measurements of a single active transcription center: 2 active copies of one-ON-state transcription have a combined ZERO-HIGH rate which can look like the LOW rate of a two-ON-state mechanism.

The model results suggest that two-ON-state Bcd and Bcd+Hb regulation can play a role in limiting RNA output variability and enhancing robustness of *eve2* patterning. To produce the same number of transcripts, the comparable one-ON-state mechanism has less time in the ON state (17% for E[1000]) than the two-ON-state mechanism (43% in E[1000] plus 10% in E[1100]), and the ON intervals are all at the HIGH initiation rate. This generates a more ‘shot’ dominated time series, with stretches of OFF interspersed with spikes of HIGH transcription. In contrast, the two-ON-state mechanism spends nearly half of its time in the E[1000] LOW rate and the HIGH rate spikes are less common. This produces a more consistent output of RNA, with a lower standard deviation than the one-ON-state mechanism. The LOW Bcd-only (E[1000]) rate insures a basal production of RNA, while Hb co-activation (E[1100] state) at the HIGH rate can boost production to maximal levels.

This work presents an approach for using stochastic modeling to analyze gene transcription time series, including from spatially-dependent expression patterns, to infer features of regulatory mechanisms. As live time series of gene transcription become available from more developmental systems, we anticipate this approach could be more generally applied for investigating simple vs. multi-state regulatory mechanisms. In the case of *eve2*, comparison of our results and the experimental time series support that the transcriptional dynamics have two distinct ON-states. The model indicates that having two-ON-states can contribute to developmental robustness by providing more consistent RNA production than a single ON-state.

## Supporting information

S1 Fig TF spatial patterns in the *eve* stripe 2 region, numbers of protein molecules against AP position. Bcd (red); Hb (black); Gt (green), early–outer, later–inner; Kr (blue), early–outer, later–inner. Bcd, Hb and early Gt and Kr profiles are adapted from FlyEx data, T1 timeclass (http://urchin.spbcas.ru/flyex/, [11]); later Gt and Kr are a ten-fold increase, 45 minutes later.
(PDF)

S2 Fig Autocorrelation for experimental time series, number of nascent transcripts. (A) Autocorrelation for the data in Fig 1B vs. time lags in minutes. (B) Autocorrelation for the data in Fig 1C. Red bands, 5% significance limits, as in Fig 3. These indicate significant autocorrelation in number of nascent transcripts at lags of 1, 2, and 3 minutes for both stripe-center nuclei.
(PDF)

S3 Fig Replicates of spatial distribution of accumulated *eve2* mRNA. 10 replicates with the same parameters and initial conditions are shown. Black line, deterministic solution, matched to *eve2* expression data; red lines, 5-minute intervals of the stochastic solutions, from 5 minutes to 45 minutes; green lines, Gt early and late; blue lines, Kr early and late. Fig 4 shows run 01311714 (closest to deterministic).
(PDF)

S4 Fig Replicates of time series at stripe-center (42%EL). From the same 10 replicates as S3 Fig. (A) Number of nascent transcripts vs. time. (B) Corresponding change-per-minute in nascent transcripts vs. time. (C) Histograms of change-per-minute for these simulations; Fig 5D is pooled from these. Fig 5A–5C show run 01311714 (closest to the experimental mean of 230 mRNA produced in nc14).
(PDF)

S5 Fig Replicates of time series at stripe edge positions. (A-C) 38%EL position, same 10 replicates as S3 and S4 Figs. (A) Number of nascent transcripts vs. time. (B) Corresponding change-per-minute in nascent transcripts vs. time. (C) Histograms of change-per-minute for these simulations; Fig 6D is pooled from these. Fig 6A–6C show run 01311714, as above. (D-F) 46%EL position, same 10 replicates. (D) Number of nascent transcripts vs. time. (E) Corresponding change-per-minute in nascent transcripts vs. time. (F) Histograms of change-per-minute for these 10 simulations; Fig 6H is pooled from these. Fig 6E–6G show run 01311714, as above.
(PDF)

S6 Fig Replicates of simulations with reduced expression from blocking Hb co-activation. (A) Spatial patterns for 10 replicates of the ZERO-LOW E[1000] state activation model, simulating reduced *eve2* expression following mutagenesis of the Hb BS. 10 replicates of the same conditions are shown. Black and red lines as in S3 Fig. Fig 7A shows run 05251604 (closest to deterministic). (B) Time series for number of nascent transcripts vs. time; (C) corresponding change-per-minute in nascent transcripts vs. time; (D) histograms of change-per-minute for these simulations; Fig 7D is pooled from these. Fig 7B–7D show run 05251604 (same as Fig 7A).
(PDF)

S7 Fig Replicates of time series for the single ON-state (simple ON-OFF) mechanism. 10 replicates with the same parameters and initial conditions, at 42%EL. (A) Number of nascent transcripts vs. time. (B) Corresponding change-per-minute in nascent transcripts vs. time. (C) Histograms of change-per-minute for these simulations; Fig 8D is pooled from these. Fig 8A–8C show run 01311724 (the closest to the experimental mean of 230 mRNA produced in nc14).
(PDF)

S8 Fig Transcription from 2 gene copies vs. 1 gene copy. (A, B) Pooled change-per-minute histograms from 10 simulations each of transcription from a single gene copy (as in Figs 4–8). (A) two-ON-state ZERO-LOW-HIGH mechanism; (B) one-ON-state ZERO-HIGH mechanism. (C, D) Pooled change-per-minute histograms from 10 simulations each of the corresponding simulations, but transcribed from 2 independent copies of the gene, each producing (on average) half of the transcripts (results shown are for the summed nuclear output). (C) two-ON-state ZERO-LOW-HIGH mechanism; (D) one-ON-state ZERO-HIGH mechanism. 2-copy transcription increases the number of ON states, e.g. ZERO-ZERO, ZERO-HIGH, AND HIGH-HIGH in (D), and 6 such combined states in (C). The highest initiation rates (HIGH-HIGH) are less often achieved with 2-copy transcription and there is an increase in states and increased occupation of low rates (blue) compared to 1-copy transcription. 2-copy transcription decreases the difference between the lowest 2 bars for the one-ON-state mechanism (arrows, B and D), making the rate distribution more like that of the two-ON-state mechanism. (A-D) are a set of simulations with the HIGH initiation rate at 27/min for 1-copy and 13.5/min for each of the copies in 2-copy.
(PDF)
