# Supplementary figures and images for: Transcriptional bursting in Drosophila development: Stochastic dynamics of eve stripe 2 expression
Source: PLoS One. 2017 Apr 24;12(4):e0176228. doi: 10.1371/journal.pone.0176228 (PMC5402966; doi:10.1371/journal.pone.0176228)

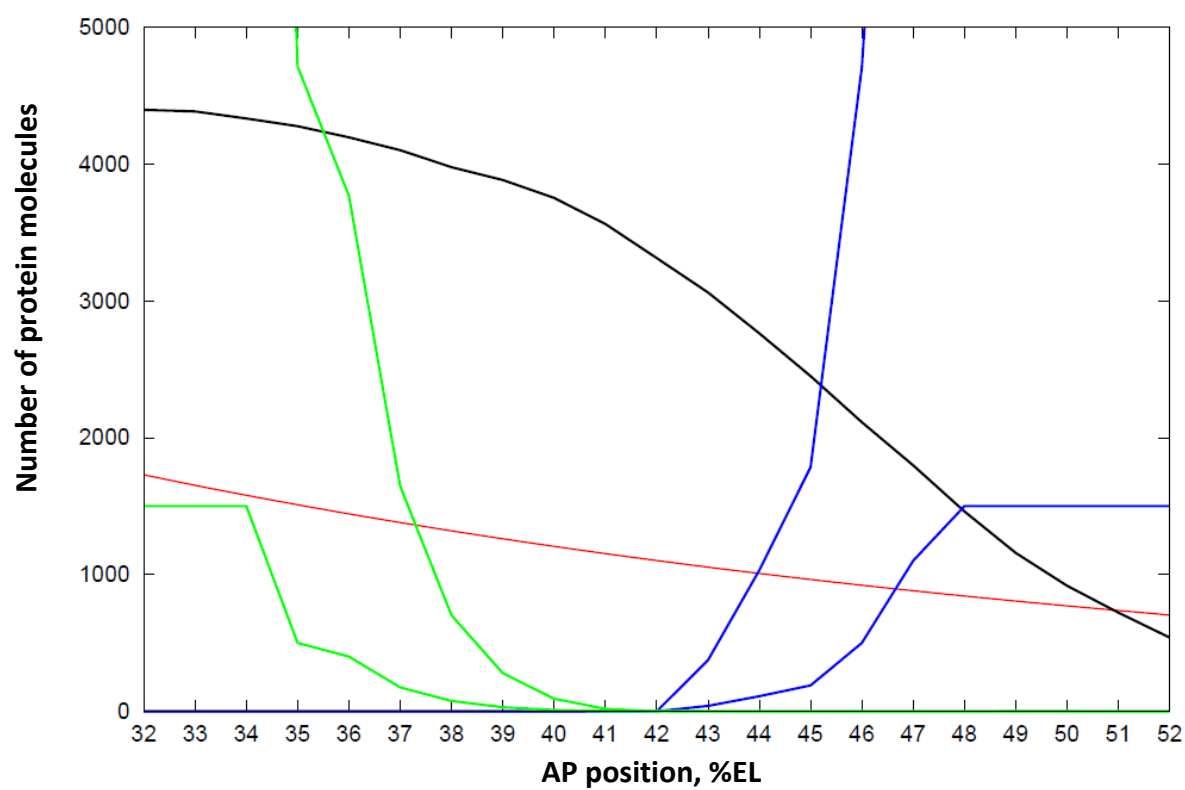

Figure S1

Supplement: S1 Fig — Bcd (red); Hb (black); Gt (green), early–outer, later–inner; Kr (blue), early–outer, later–inner. Bcd, Hb and early Gt and Kr profiles are adapted from FlyEx data, T1 timeclass (http://urchin.spbcas.ru/flyex/, [11]); later Gt and Kr are a ten-fold increase, 45 minutes later. (PDF) [file pone.0176228.s001.pdf]

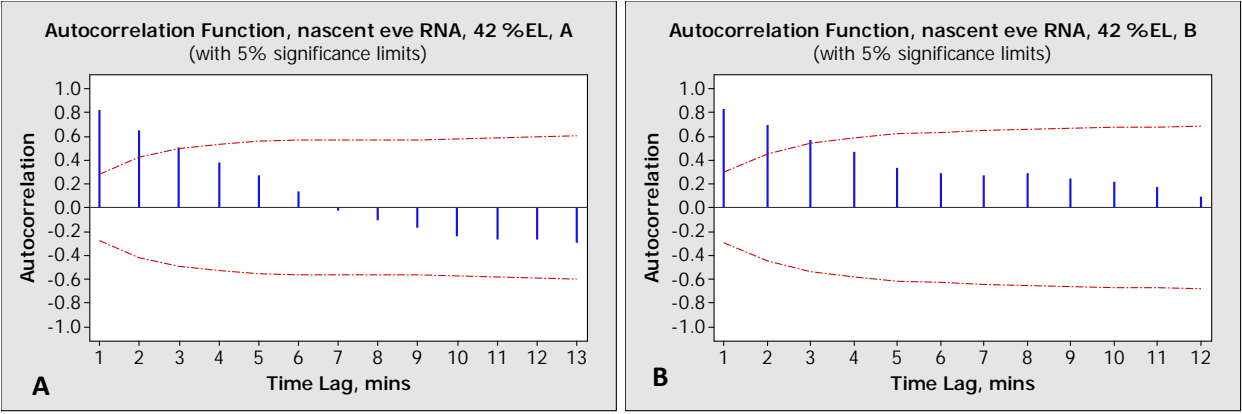

Figure S2

Supplement: S2 Fig — (A) Autocorrelation for the data in Fig 1B vs. time lags in minutes. (B) Autocorrelation for the data in Fig 1C. Red bands, 5% significance limits, as in Fig 3. These indicate significant autocorrelation in number of nascent transcripts at lags of 1, 2, and 3 minutes for both stripe-center nuclei. (PDF) [file pone.0176228.s002.pdf]

Figure S3

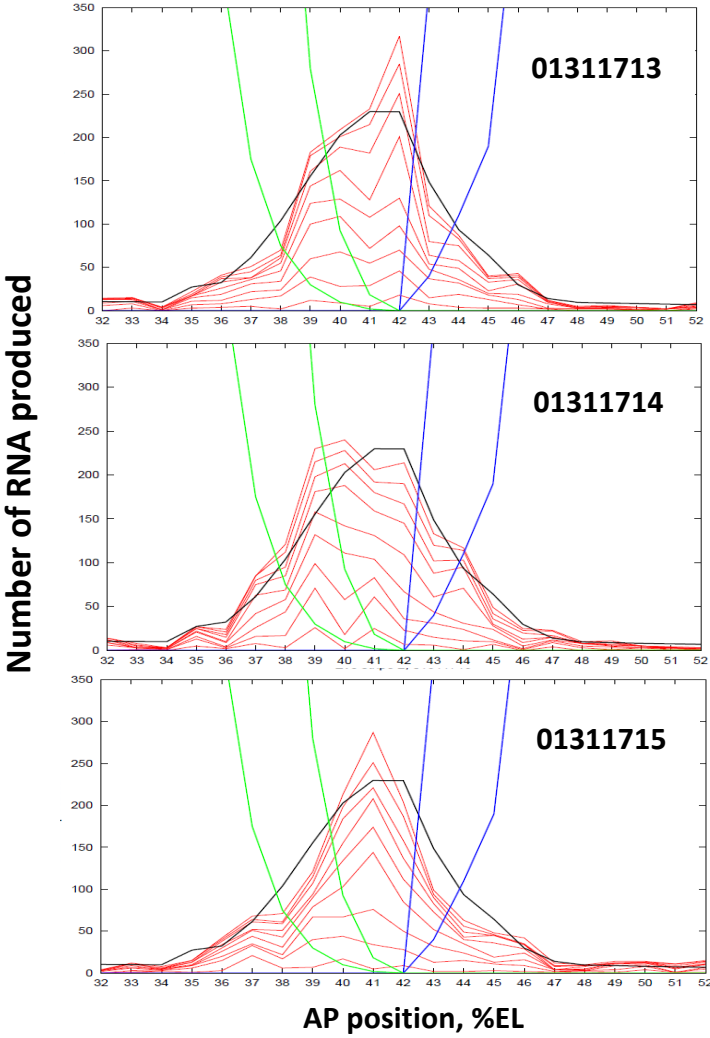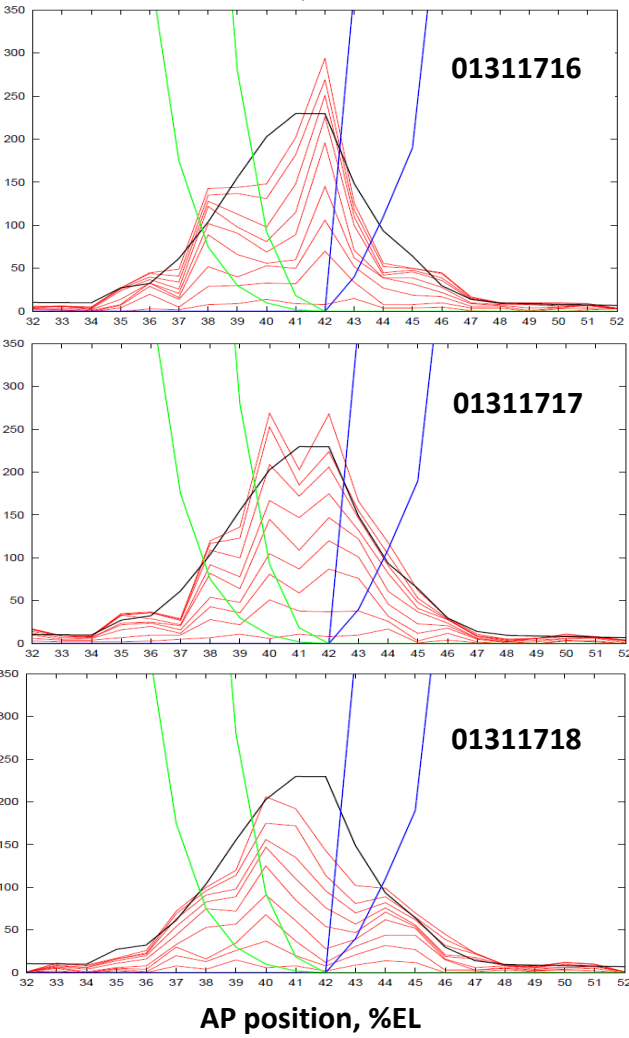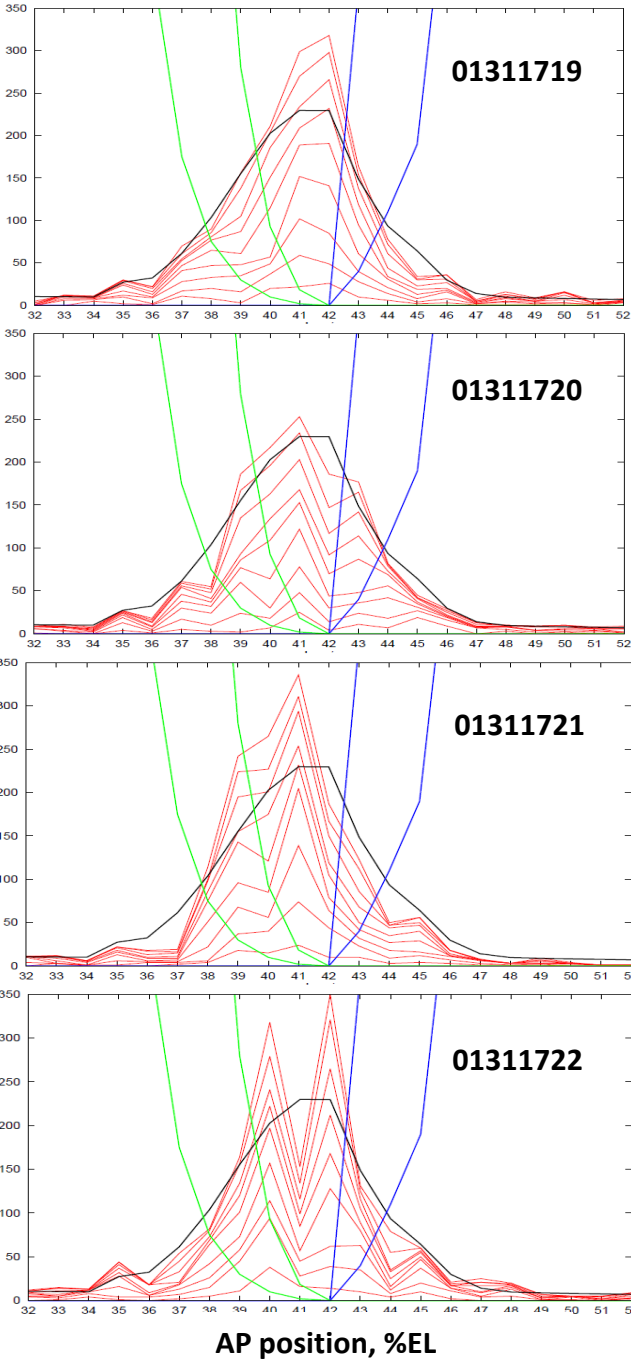

Supplement: S3 Fig — 10 replicates with the same parameters and initial conditions are shown. Black line, deterministic solution, matched to eve2 expression data; red lines, 5-minute intervals of the stochastic solutions, from 5 minutes to 45 minutes; green lines, Gt early and late; blue lines, Kr early and late. Fig 4 shows run 01311714 (closest to deterministic). (PDF) [file pone.0176228.s003.pdf]

nascent eve RNA

**Figure S4A**

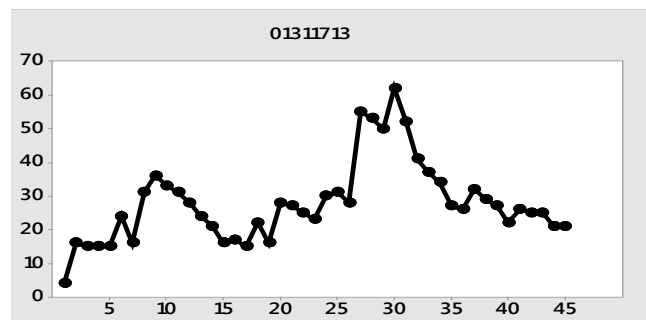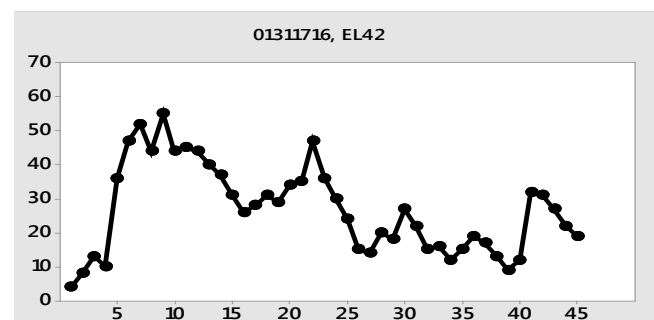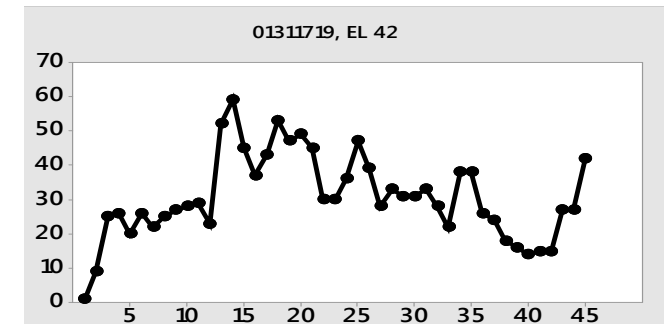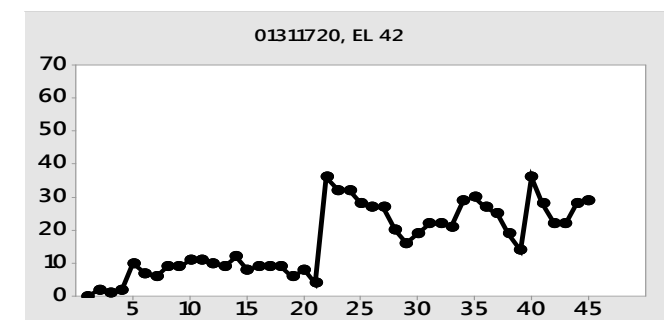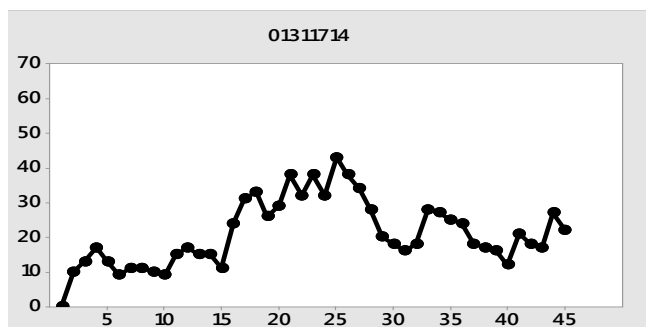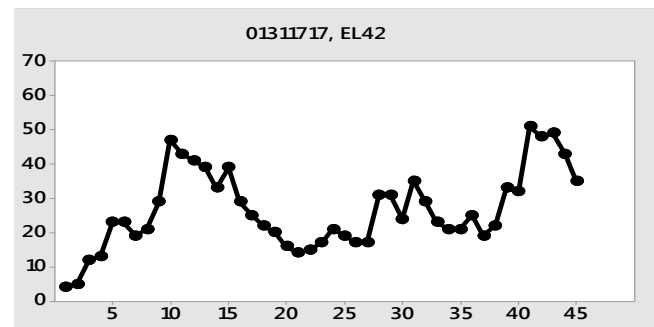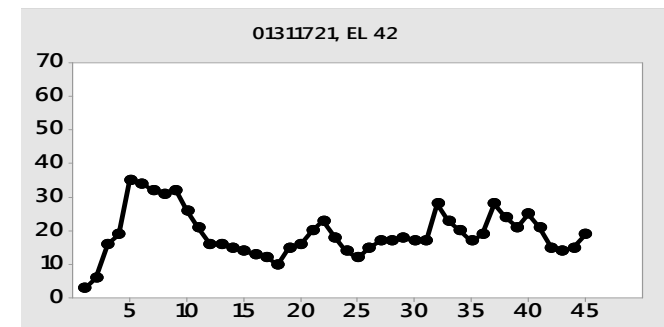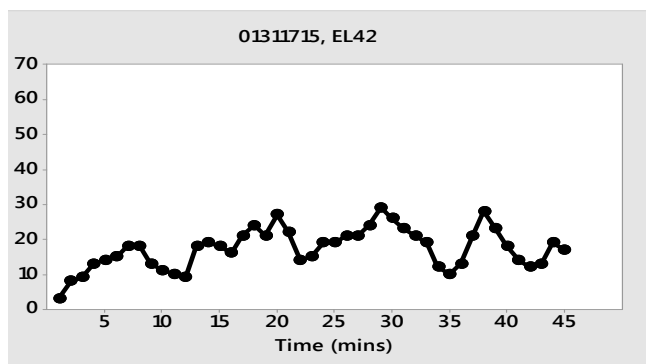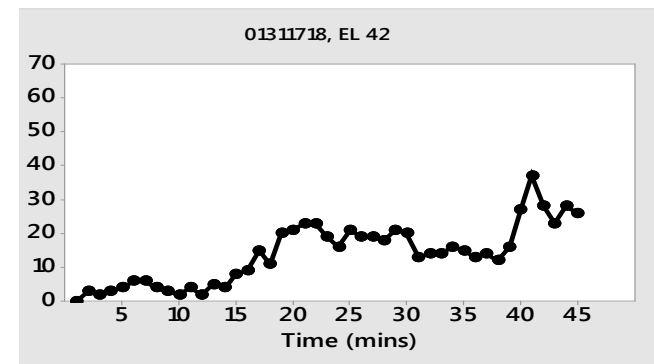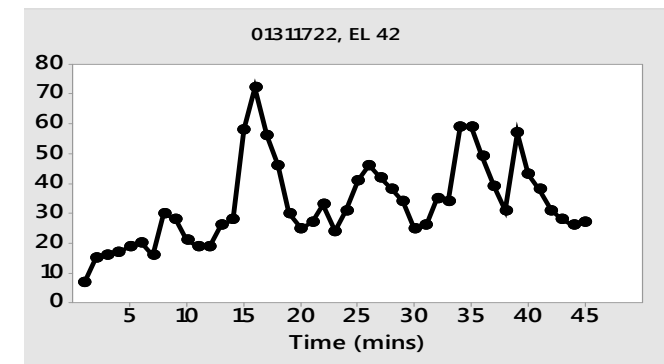

change per minute

**Figure S4B**

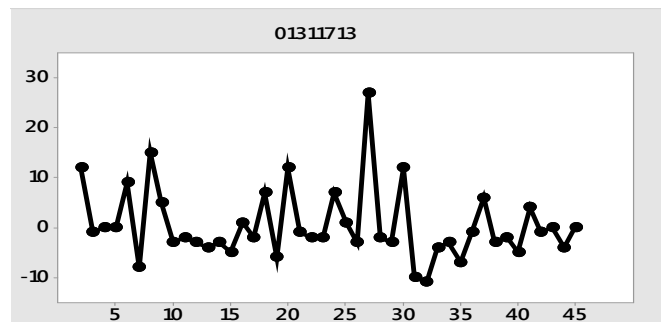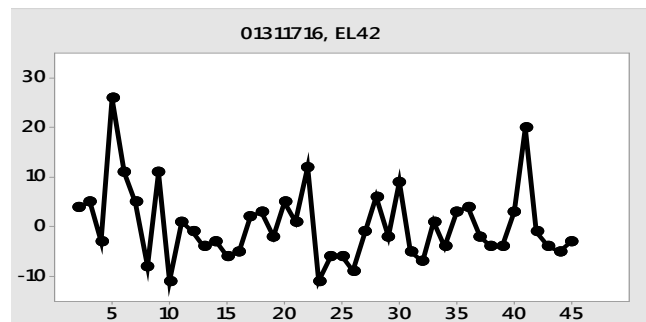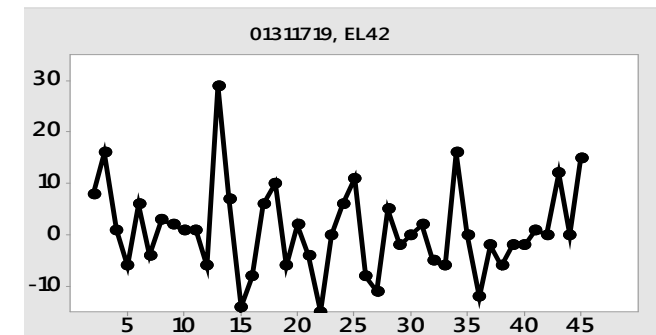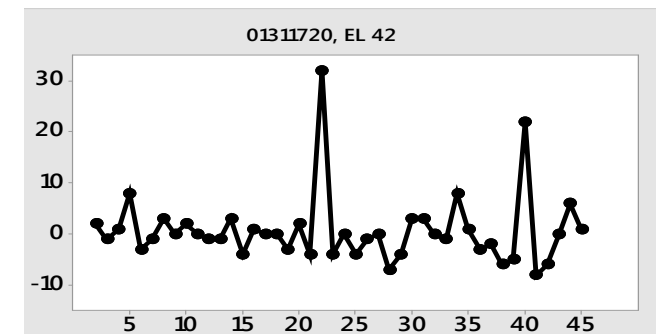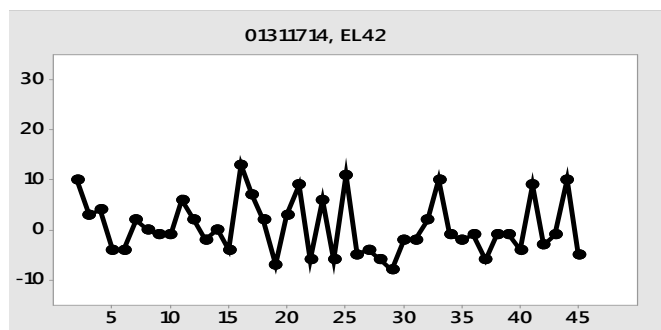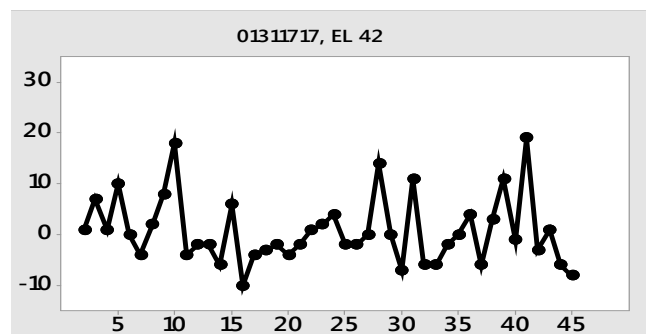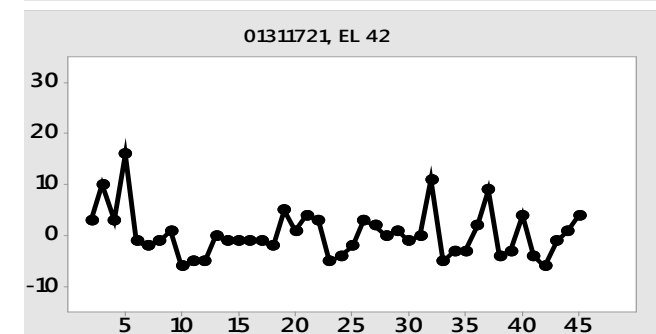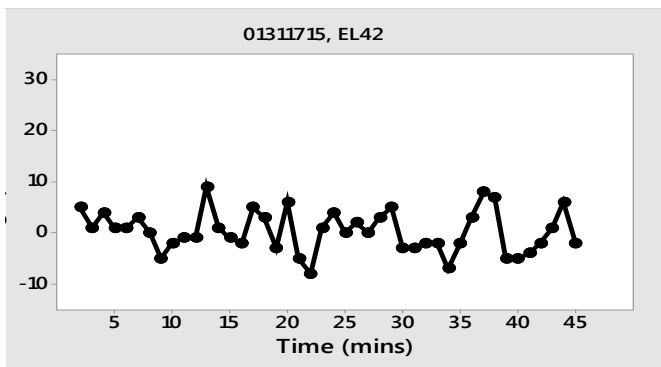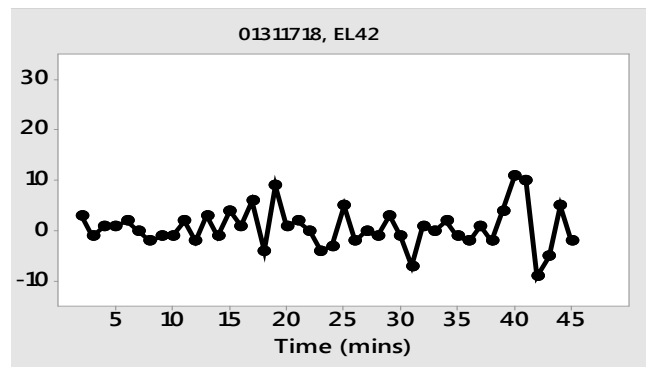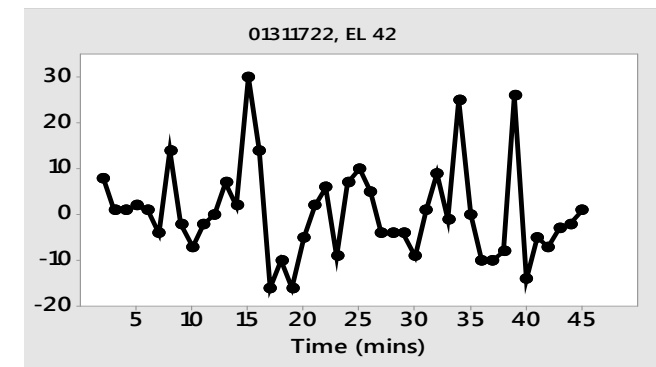

Figure S4C

change per minute

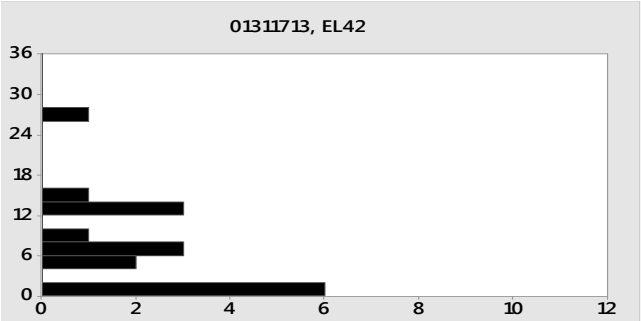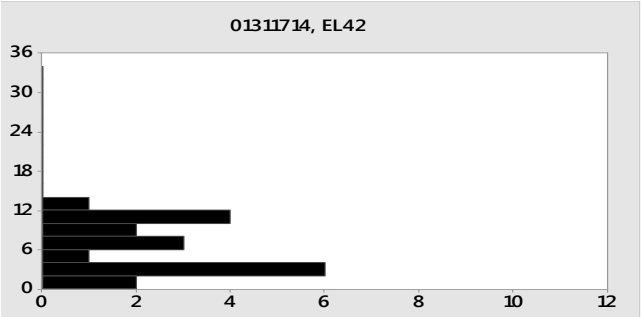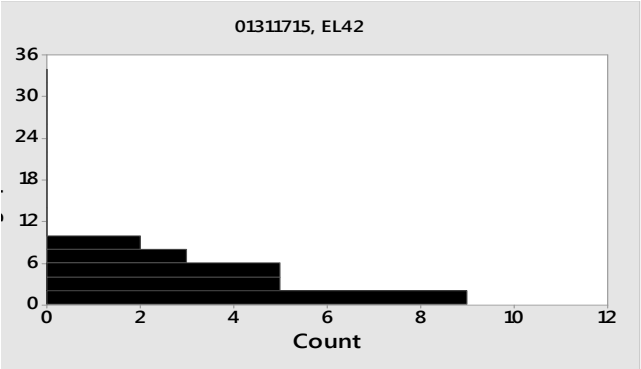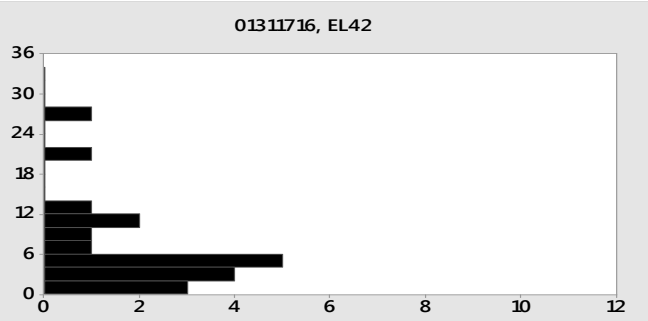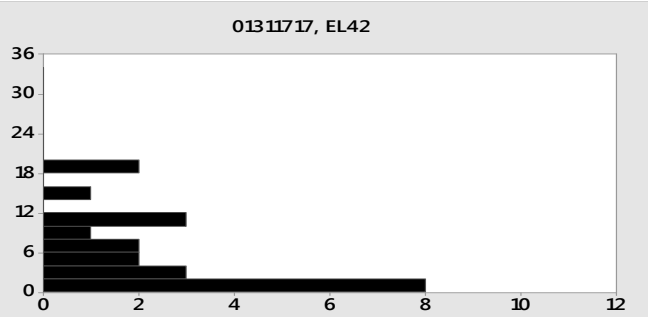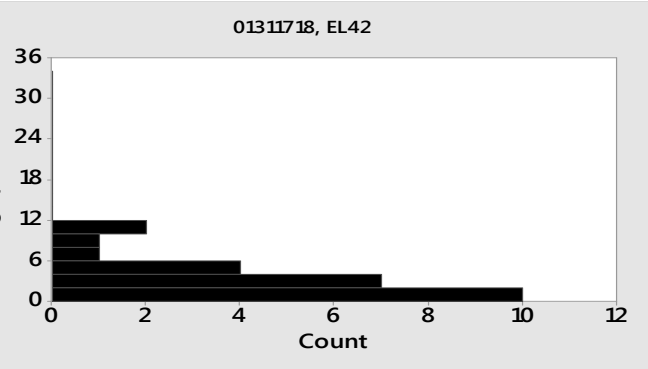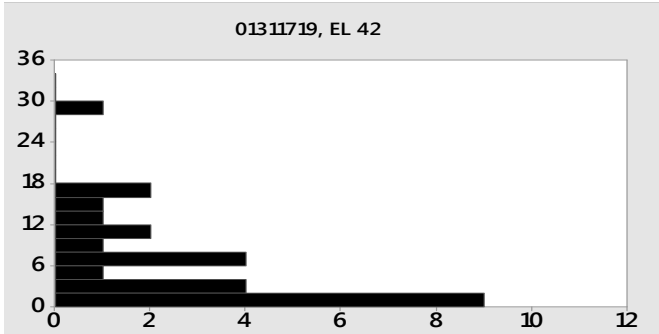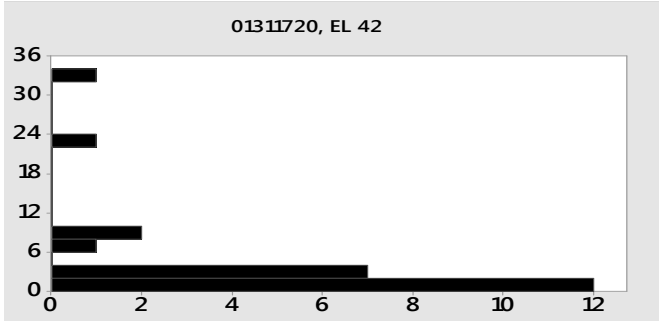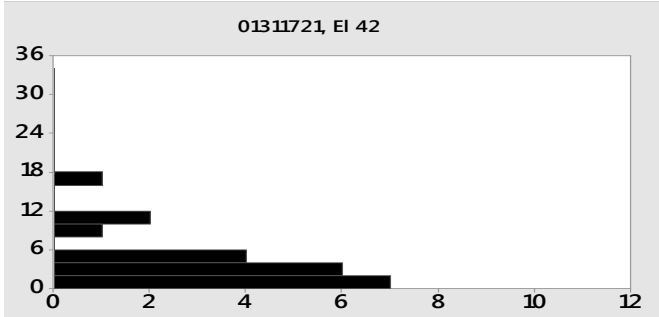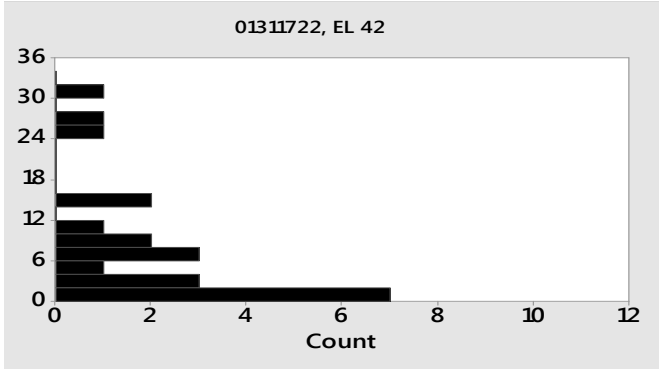

Supplement: S4 Fig — From the same 10 replicates as S3 Fig. (A) Number of nascent transcripts vs. time. (B) Corresponding change-per-minute in nascent transcripts vs. time. (C) Histograms of change-per-minute for these simulations; Fig 5D is pooled from these. Fig 5A–5C show run 01311714 (closest to the experimental mean of 230 mRNA produced in nc14). (PDF) [file pone.0176228.s004.pdf]

**Figure S7A**

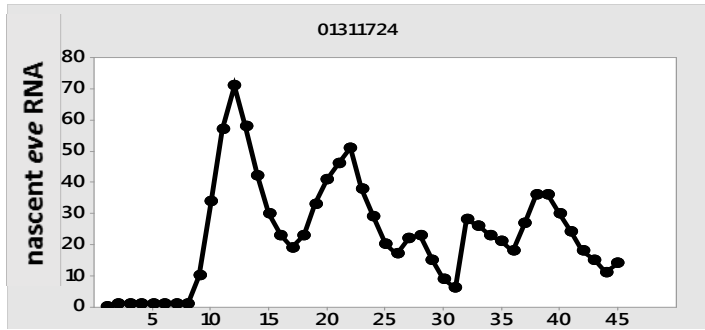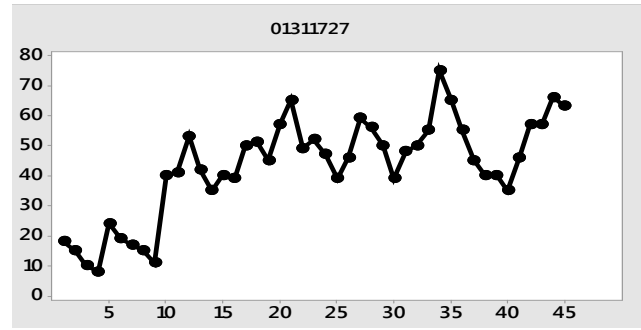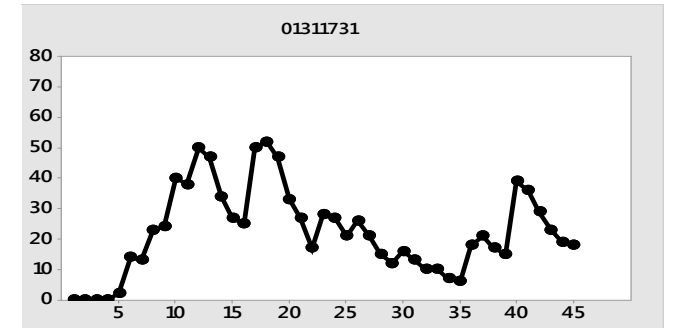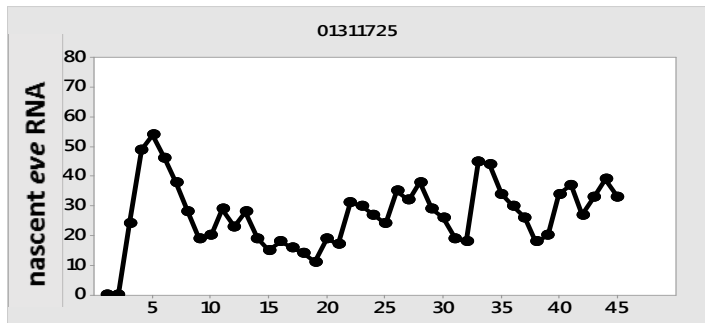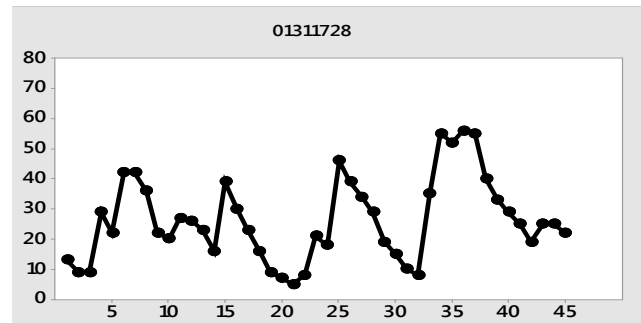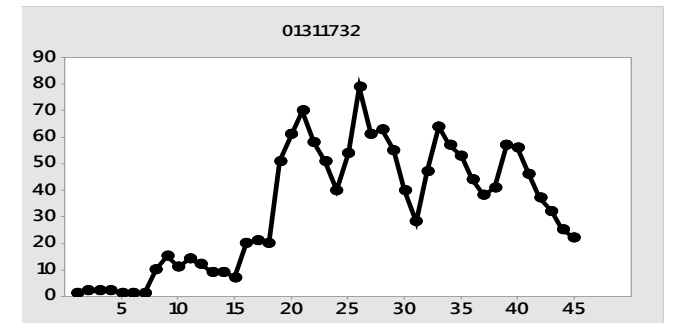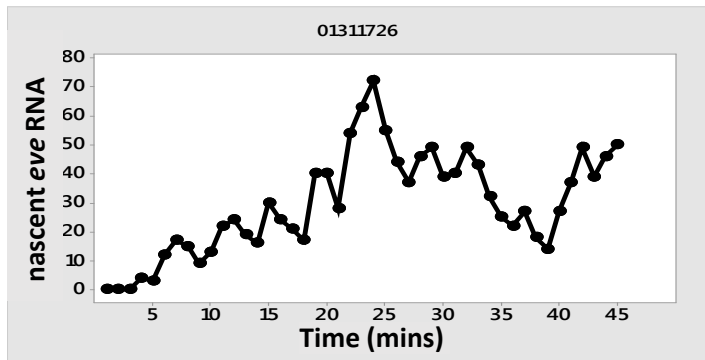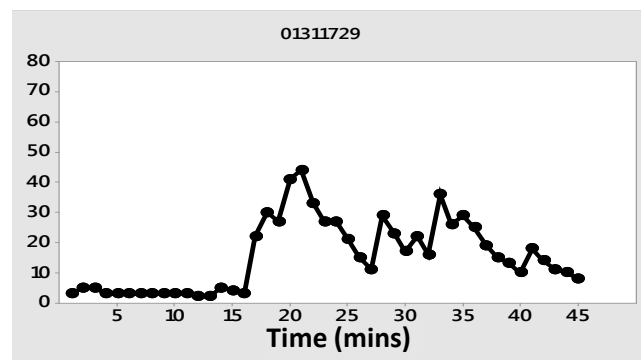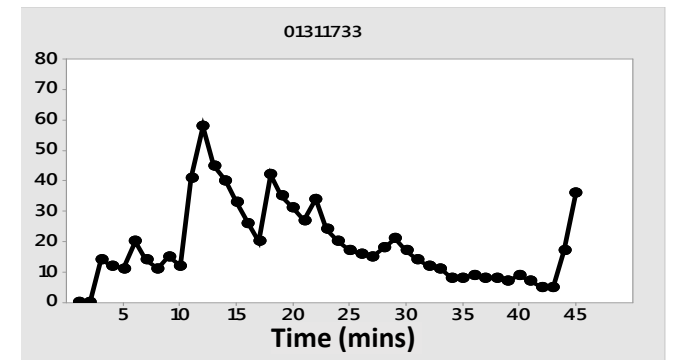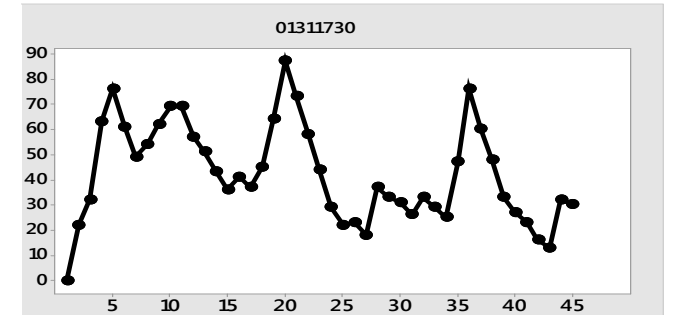

**Figure S7B**

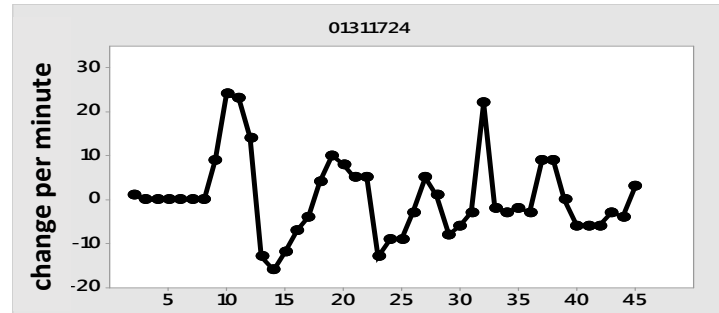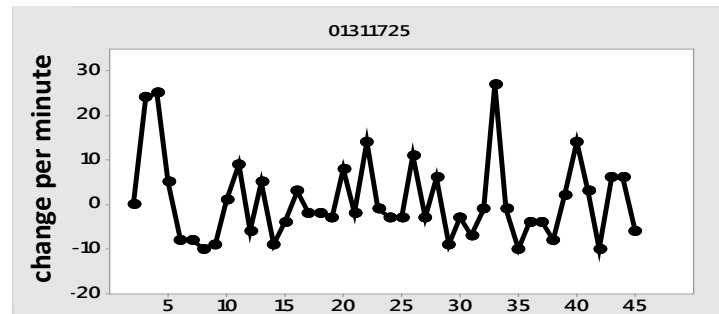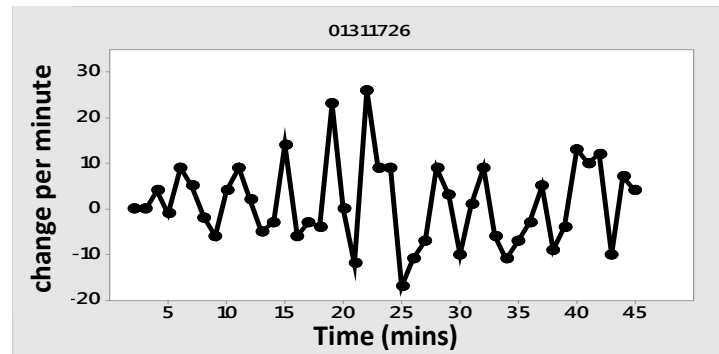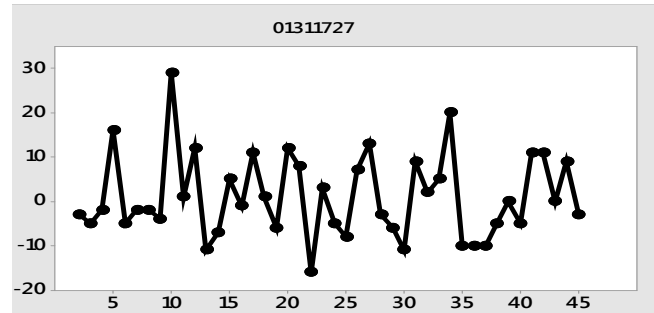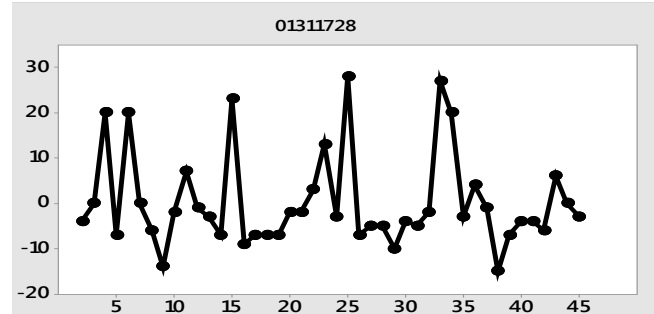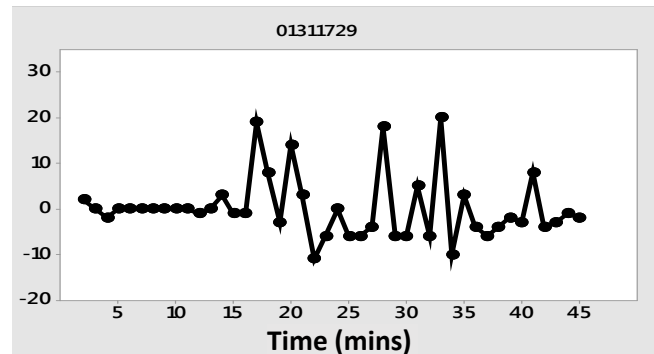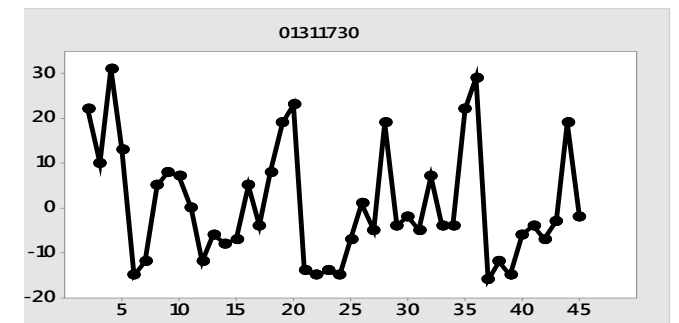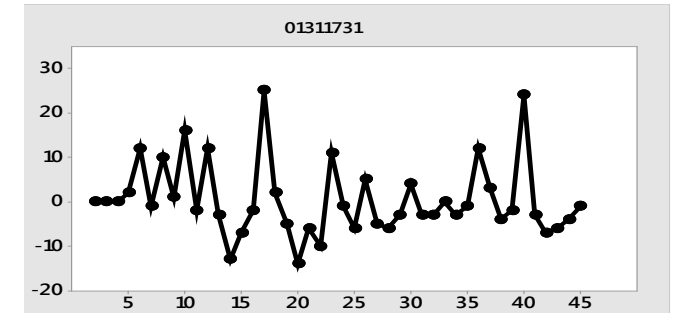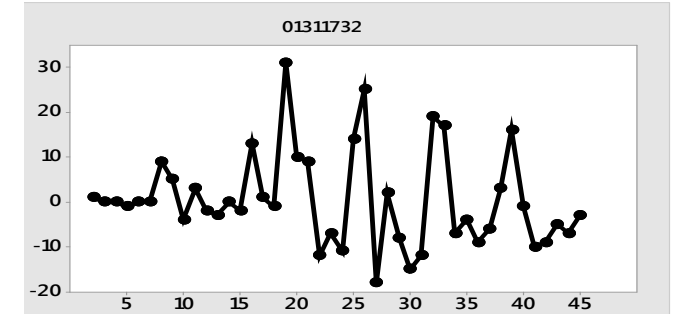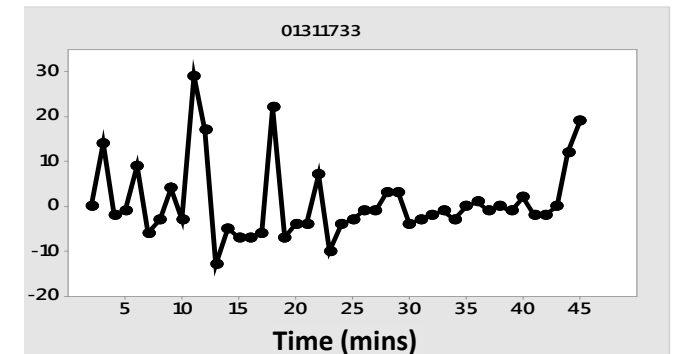

Figure S7C

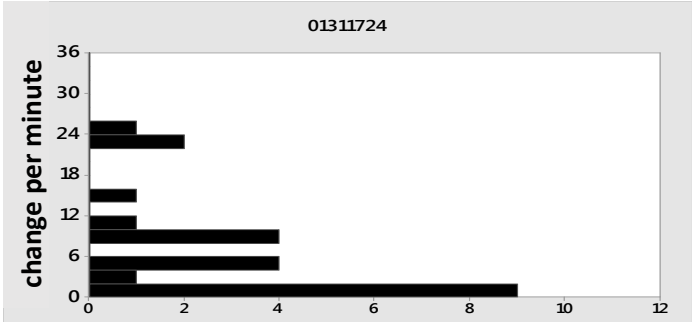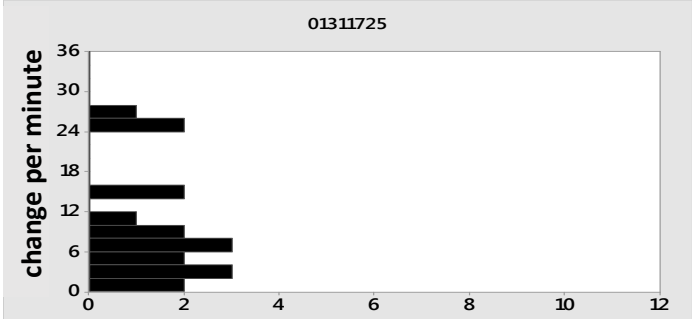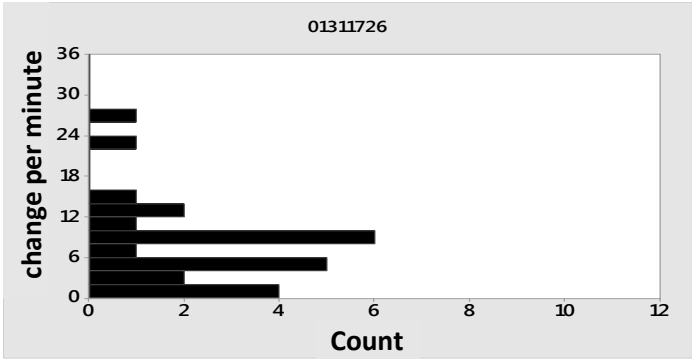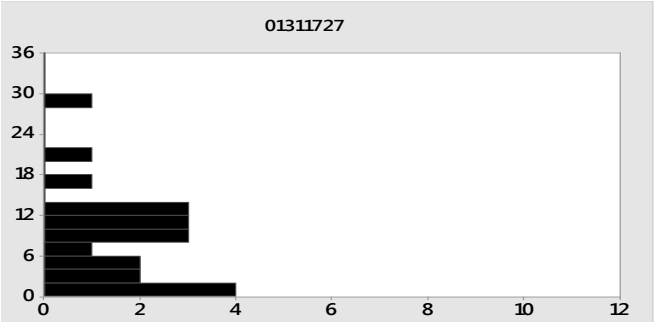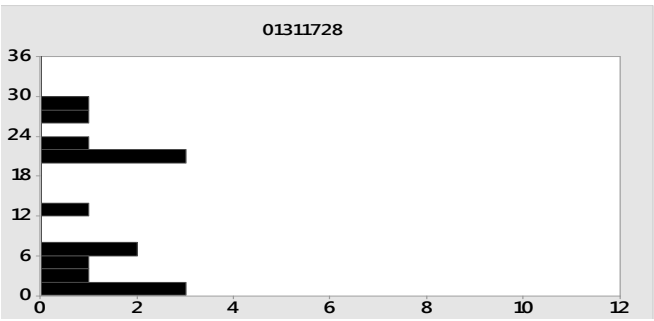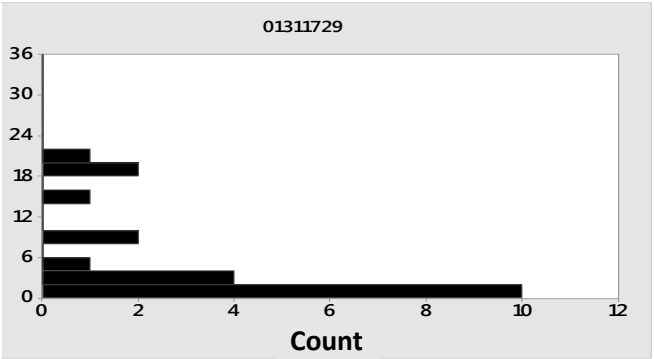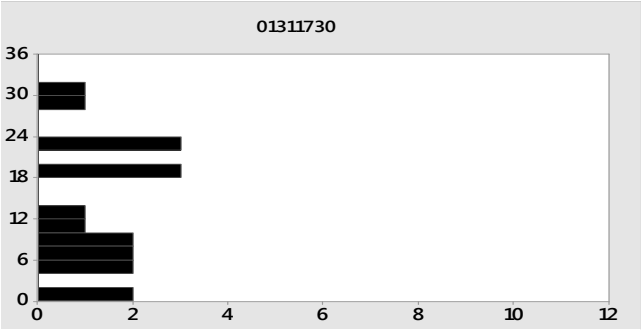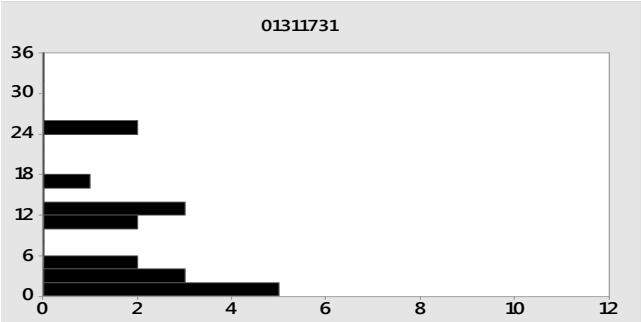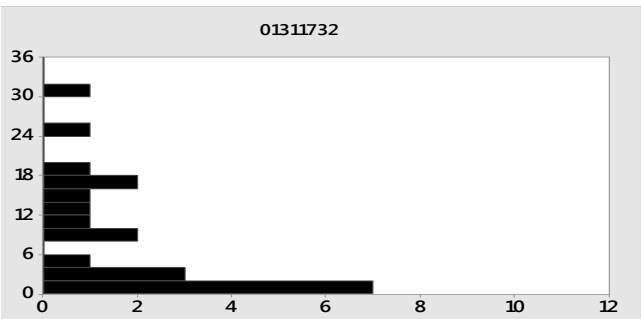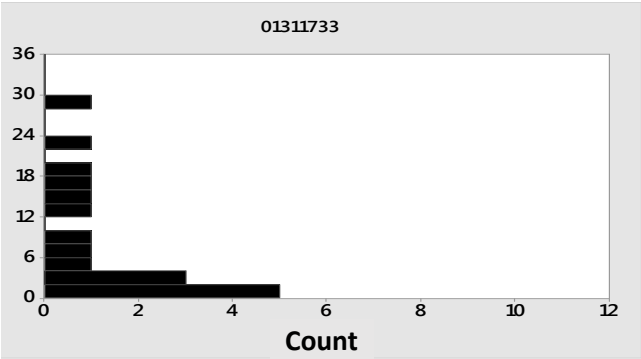

Supplement: S7 Fig — 10 replicates with the same parameters and initial conditions, at 42%EL. (A) Number of nascent transcripts vs. time. (B) Corresponding change-per-minute in nascent transcripts vs. time. (C) Histograms of change-per-minute for these simulations; Fig 8D is pooled from these. Fig 8A–8C show run 01311724 (the closest to the experimental mean of 230 mRNA produced in nc14). (PDF) [file pone.0176228.s007.pdf]

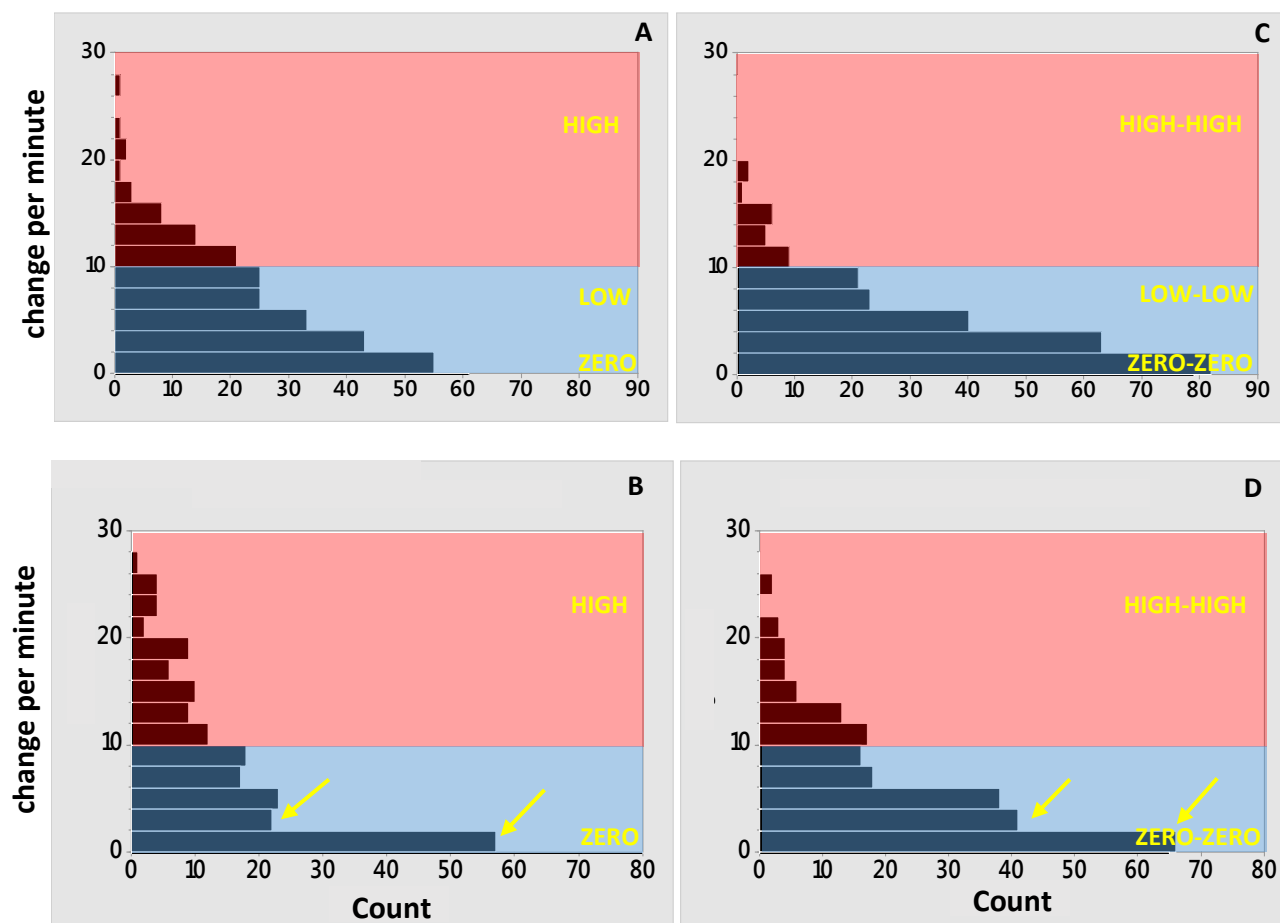

Supplement: S8 Fig — (A, B) Pooled change-per-minute histograms from 10 simulations each of transcription from a single gene copy (as in Figs 4–8). (A) two-ON-state ZERO-LOW-HIGH mechanism; (B) one-ON-state ZERO-HIGH mechanism. (C, D) Pooled change-per-minute histograms from 10 simulations each of the corresponding simulations, but transcribed from 2 independent copies of the gene, each producing (on average) half of the transcripts (results shown are for the summed nuclear output). (C) two-ON-state ZERO-LOW-HIGH mechanism; (D) one-ON-state ZERO-HIGH mechanism. 2-copy transcription increases the number of ON states, e.g. ZERO-ZERO, ZERO-HIGH, AND HIGH-HIGH in (D), and 6 such combined states in (C). The highest initiation rates (HIGH-HIGH) are less often achieved with 2-copy transcription and there is an increase in states and increased occupation of low rates (blue) compared to 1-copy transcription. 2-copy transcription decreases the difference between the lowest 2 bars for the one-ON-state mechanism (arrows, B and D), making the rate distribution more like that of the two-ON-state mechanism. (A-D) are a set of simulations with the HIGH initiation rate at 27/min for 1-copy and 13.5/min for each of the copies in 2-copy. (PDF) [file pone.0176228.s008.pdf]
