# Supplementary material for: Transcriptional bursting in Drosophila development: Stochastic dynamics of eve stripe 2 expression
Source: PLoS One. 2017 Apr 24;12(4):e0176228. doi: 10.1371/journal.pone.0176228 (PMC5402966; doi:10.1371/journal.pone.0176228)
Supplement: S5 Fig — (A-C) 38%EL position, same 10 replicates as S3 and S4 Figs. (A) Number of nascent transcripts vs. time. (B) Corresponding change-per-minute in nascent transcripts vs. time. (C) Histograms of change-per-minute for these simulations; Fig 6D is pooled from these. Fig 6A–6C show run 01311714, as above. (D-F) 46%EL position, same 10 replicates. (D) Number of nascent transcripts vs. time. (E) Corresponding change-per-minute in nascent transcripts vs. time. (F) Histograms of change-per-minute for these 10 simulations; Fig 6H is pooled from these. Fig 6E–6G show run 01311714, as above. (PDF) [file pone.0176228.s005.pdf]

Figure S5A

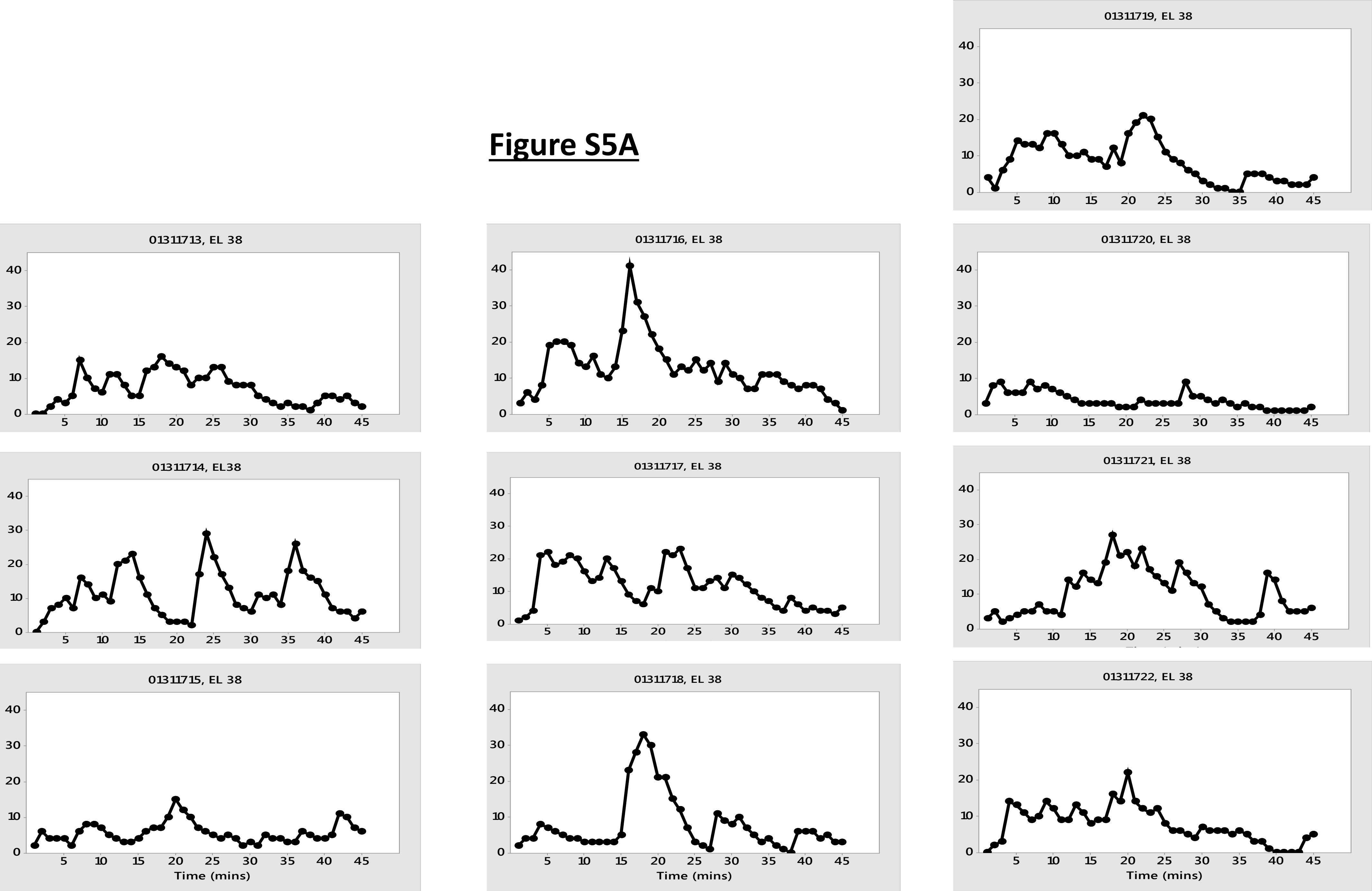
01311719, EL 38
01311713, EL 38
01311716, EL 38
01311720, EL 38
01311714, EL38
01311717, EL 38
01311721, EL 38
01311715, EL 38
01311718, EL 38
01311722, EL 38
Time (mins)
nascent *eve* RNA

Figure S5B

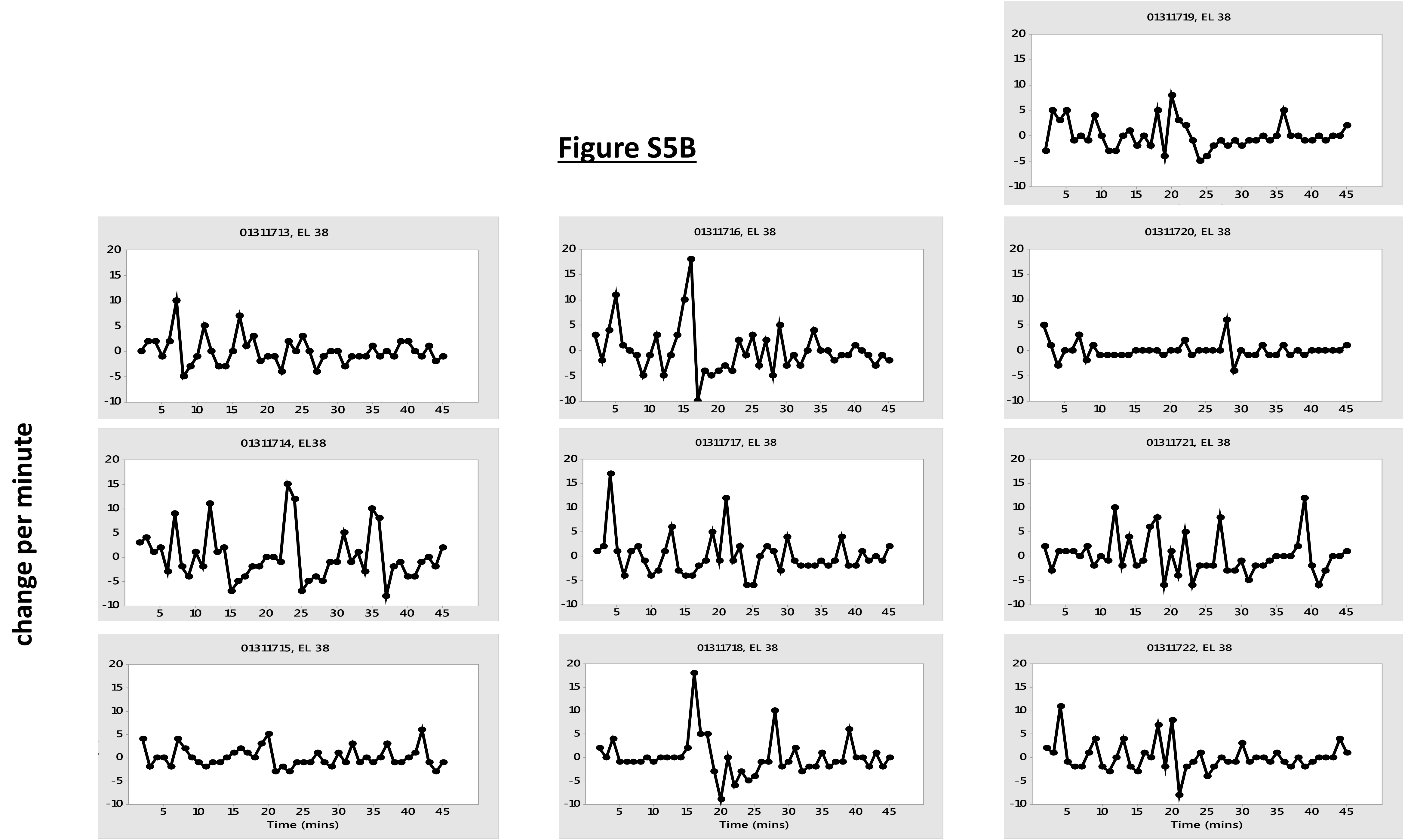
change per minute
01311719, EL 38
01311713, EL 38
01311716, EL 38
01311720, EL 38
01311714, EL38
01311717, EL 38
01311721, EL 38
01311715, EL 38
01311718, EL 38
01311722, EL 38
Time (mins)
Time (mins)
Time (mins)

Figure S5C

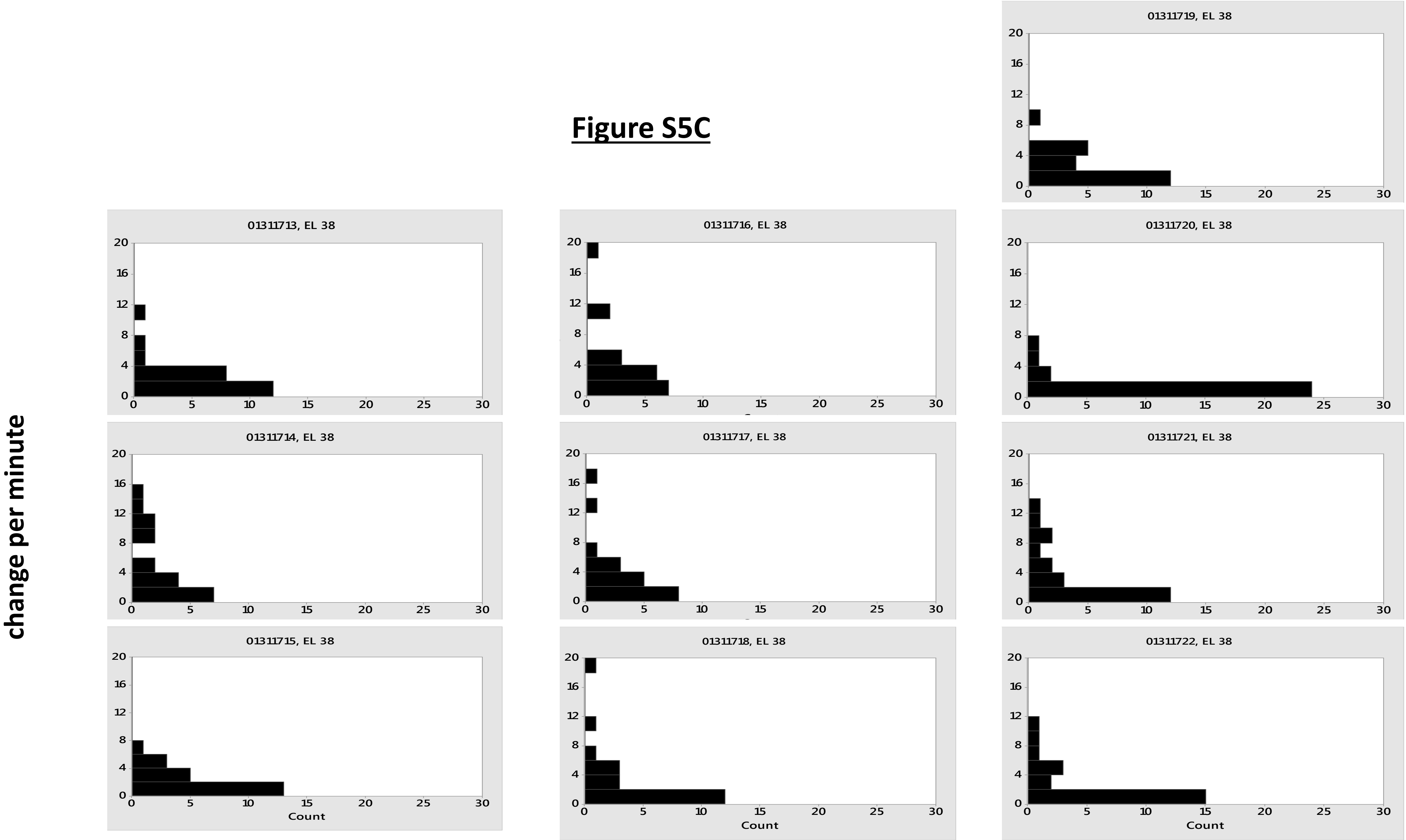
change per minute
01311719, EL 38
01311713, EL 38
01311716, EL 38
01311720, EL 38
01311714, EL 38
01311717, EL 38
01311721, EL 38
01311715, EL 38
01311718, EL 38
01311722, EL 38
Count
Count
Count

Figure S5D

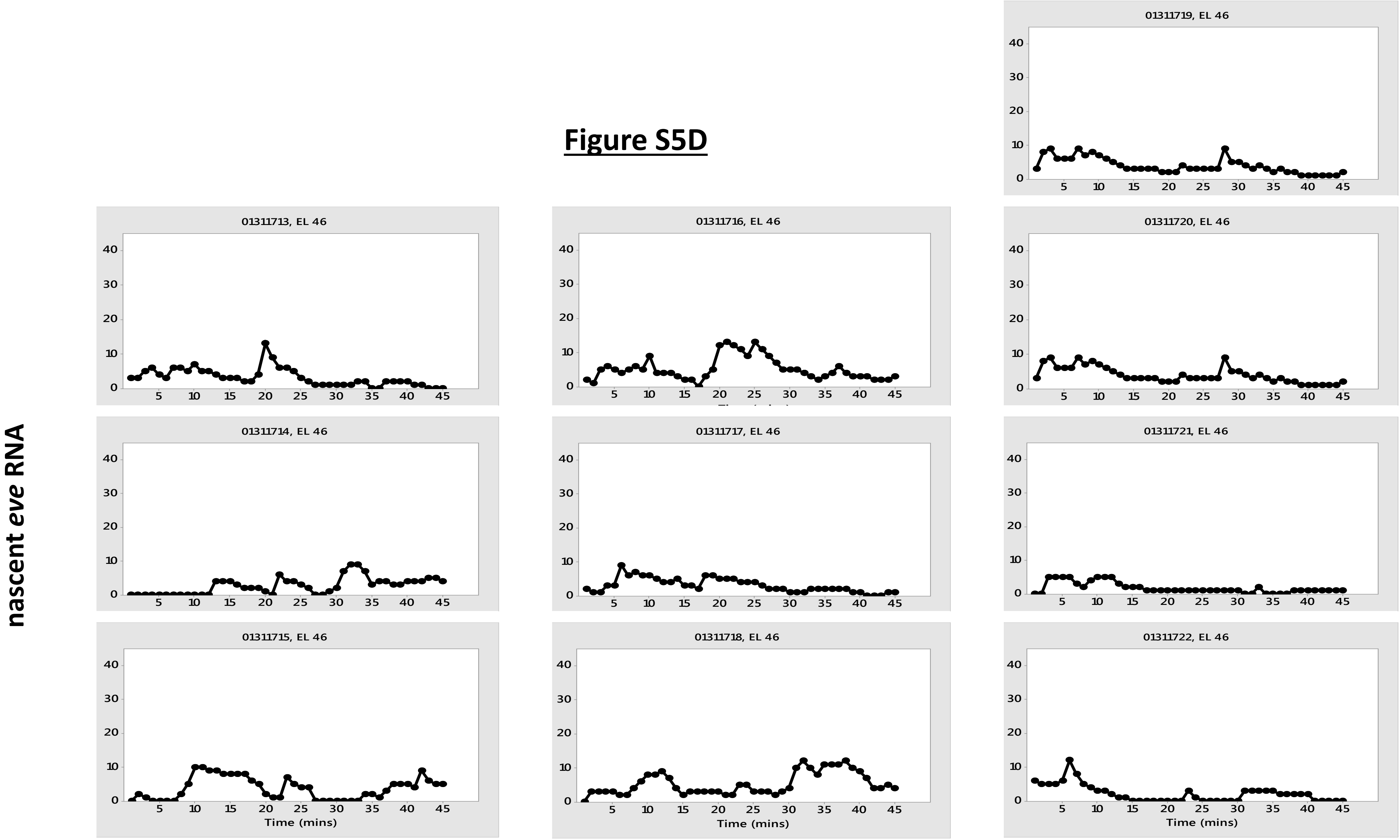
nascent eve RNA
01311719, EL 46
01311713, EL 46
01311716, EL 46
01311720, EL 46
01311714, EL 46
01311717, EL 46
01311721, EL 46
01311715, EL 46
01311718, EL 46
01311722, EL 46
Time (mins)
Time (mins)
Time (mins)

Figure S5E

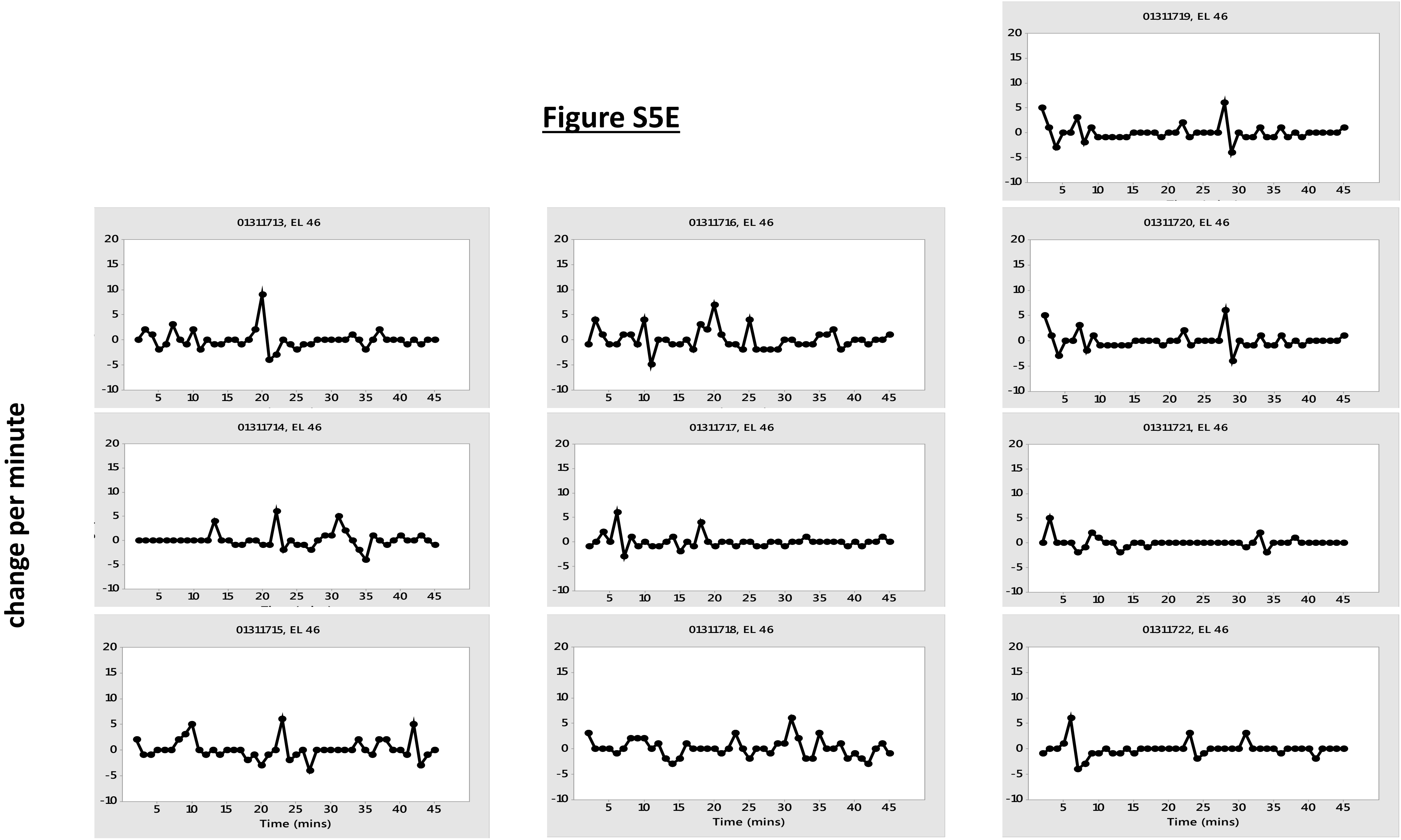

change per minute
01311719, EL 46
01311713, EL 46
01311716, EL 46
01311720, EL 46
01311714, EL 46
01311717, EL 46
01311721, EL 46
01311715, EL 46
01311718, EL 46
01311722, EL 46
Time (mins)
Time (mins)
Time (mins)

Figure S5F

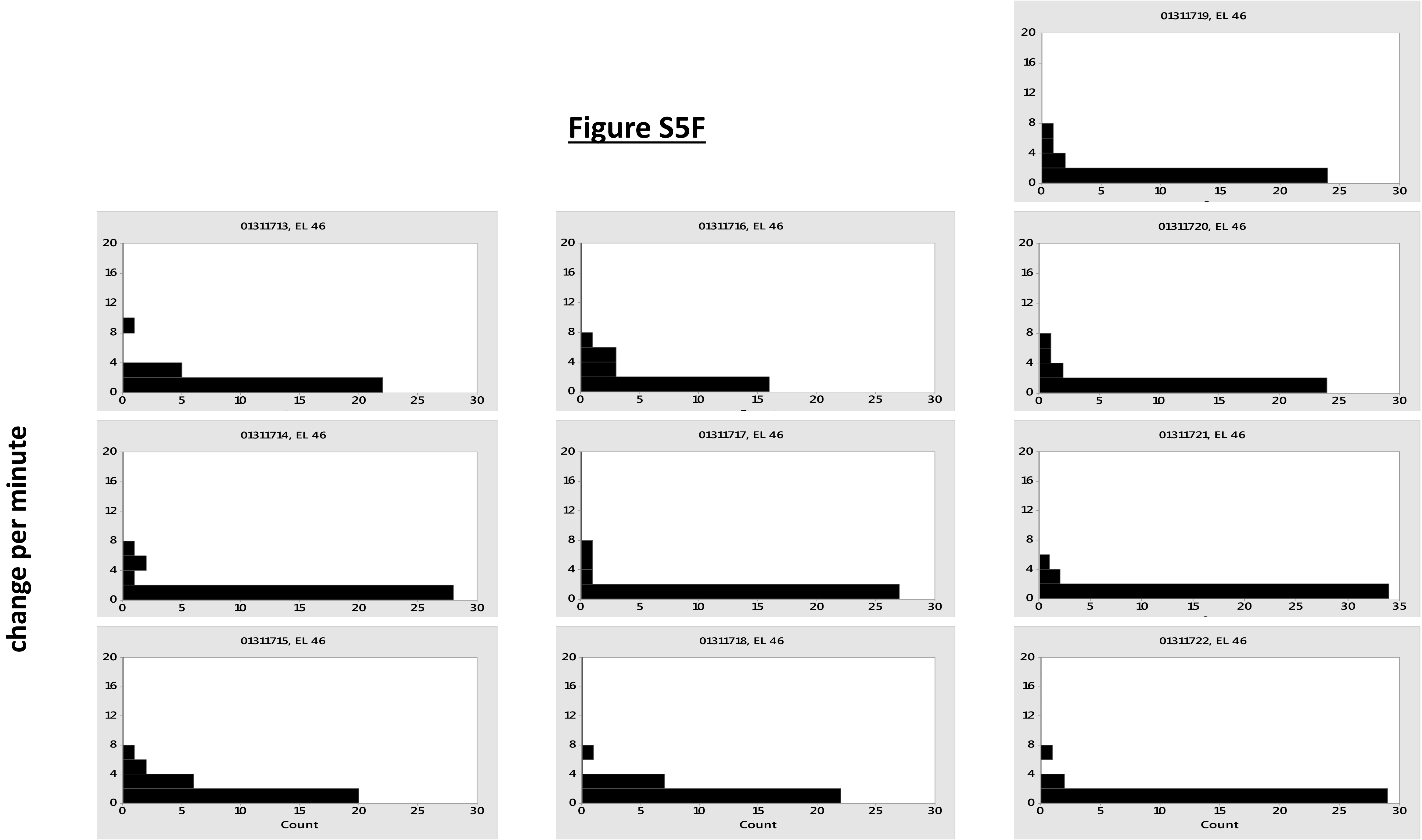

change per minute
01311719, EL 46
01311713, EL 46
01311716, EL 46
01311720, EL 46
01311714, EL 46
01311717, EL 46
01311721, EL 46
01311715, EL 46
01311718, EL 46
01311722, EL 46
0 4 8 12 16 20
0 5 10 15 20 25 30 35
Count
