# Supplementary material for: Transcriptional bursting in Drosophila development: Stochastic dynamics of eve stripe 2 expression
Source: PLoS One. 2017 Apr 24;12(4):e0176228. doi: 10.1371/journal.pone.0176228 (PMC5402966; doi:10.1371/journal.pone.0176228)
Supplement: S6 Fig — (A) Spatial patterns for 10 replicates of the ZERO-LOW E[1000] state activation model, simulating reduced eve2 expression following mutagenesis of the Hb BS. 10 replicates of the same conditions are shown. Black and red lines as in S3 Fig. Fig 7A shows run 05251604 (closest to deterministic). (B) Time series for number of nascent transcripts vs. time; (C) corresponding change-per-minute in nascent transcripts vs. time; (D) histograms of change-per-minute for these simulations; Fig 7D is pooled from these. Fig 7B–7D show run 05251604 (same as Fig 7A). (PDF) [file pone.0176228.s006.pdf]

Figure S6A

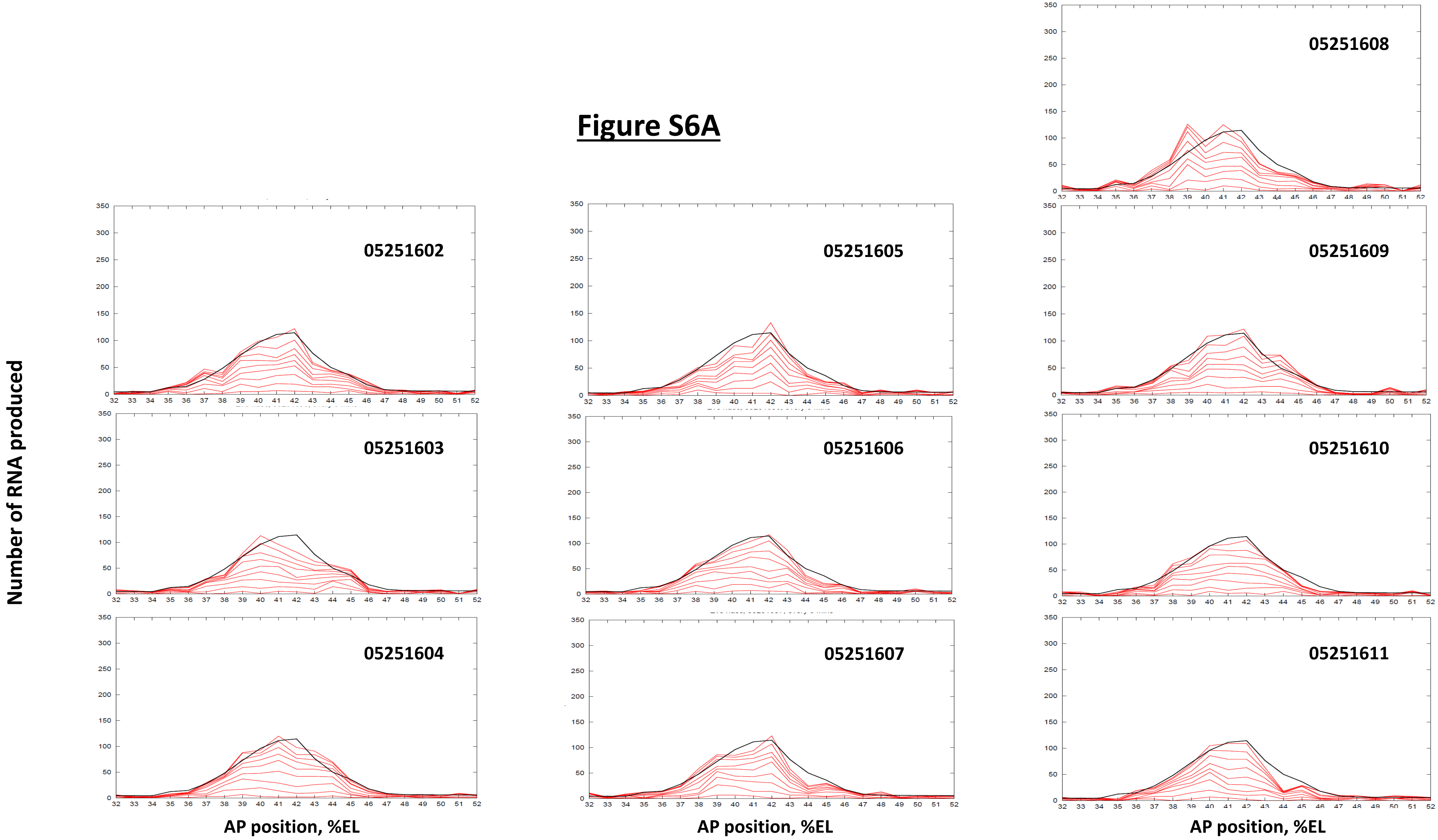
05251608
05251602
05251605
05251609
05251603
05251606
05251610
05251604
05251607
05251611
Number of RNA produced
AP position, %EL
AP position, %EL
AP position, %EL

Figure S6B

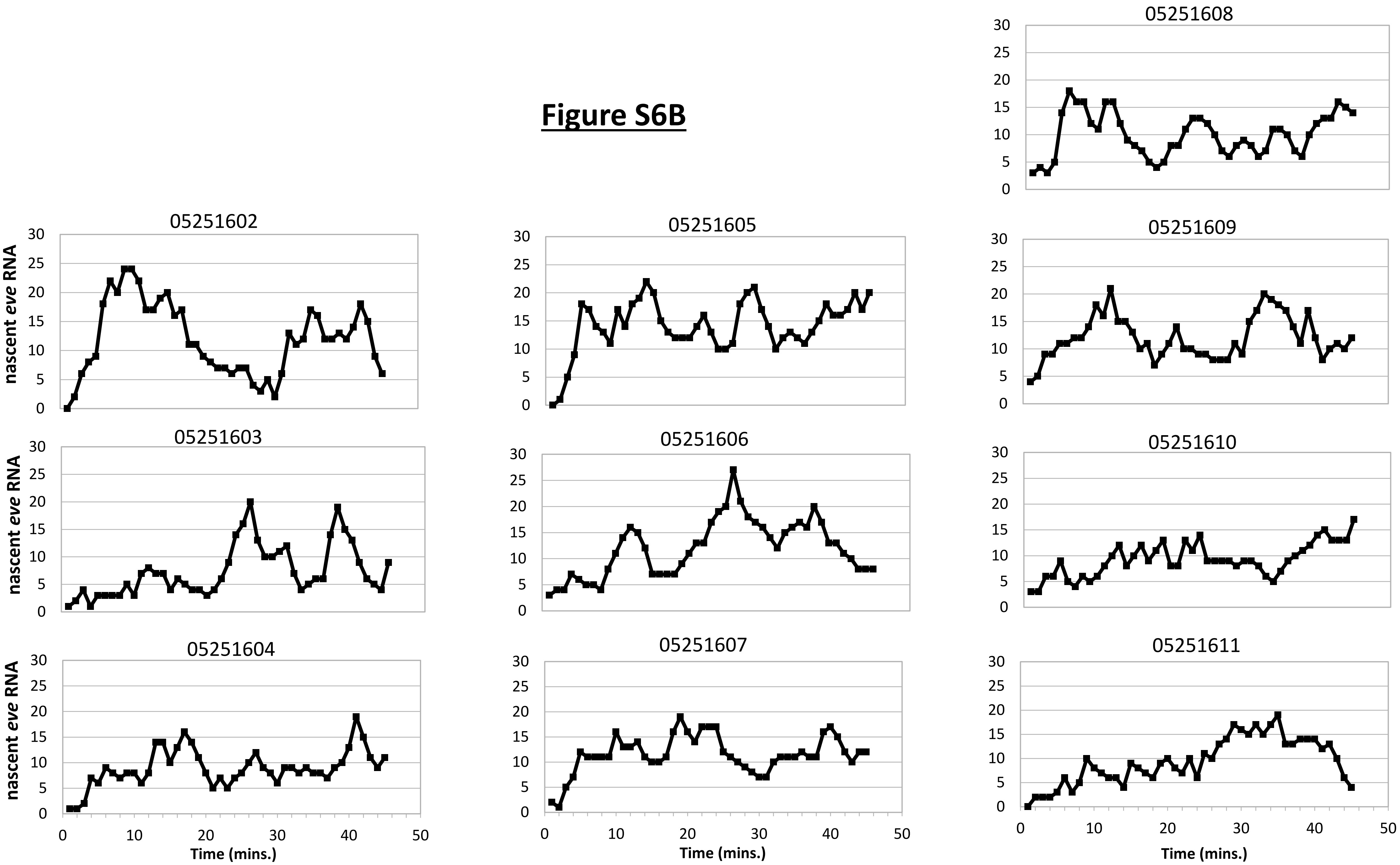
05251608
05251602
05251605
05251609
05251603
05251606
05251610
05251604
05251607
05251611
nascent *eve* RNA
Time (mins.)
0
5
10
15
20
25
30
40
50

Figure S6C

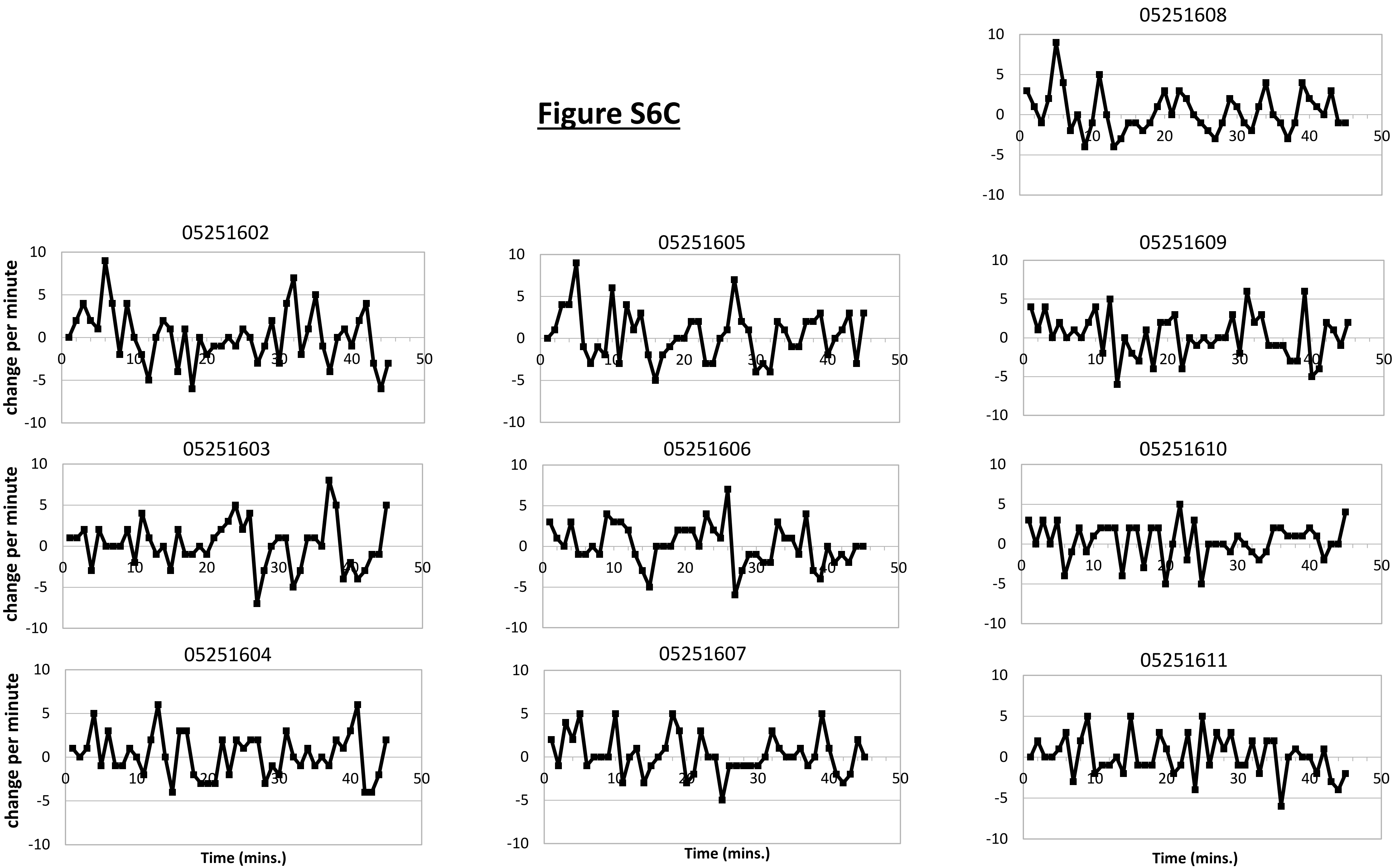
05251608
05251602
05251605
05251609
05251603
05251606
05251610
05251604
05251607
05251611
change per minute
Time (mins.)

Figure S6D

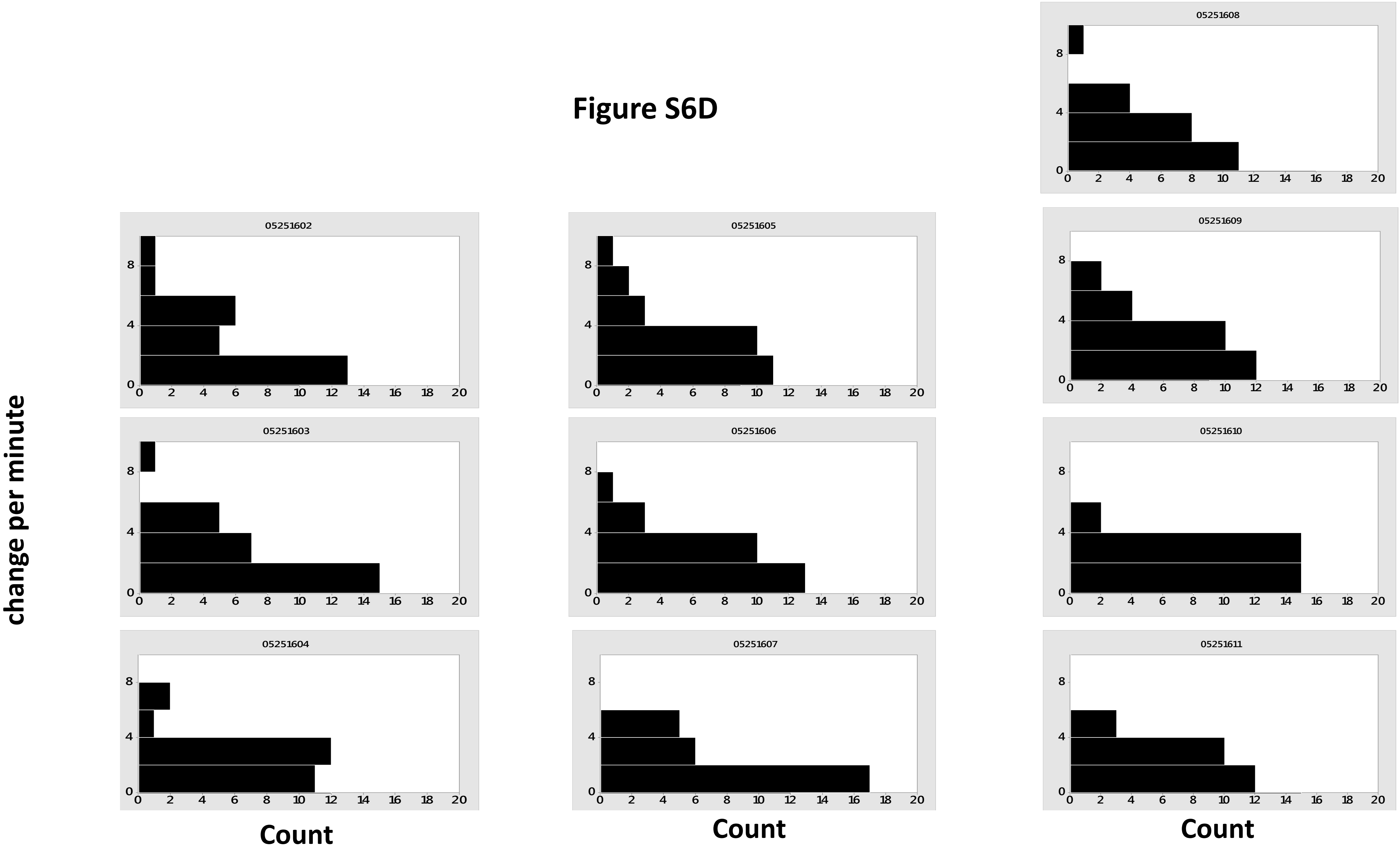
05251608
05251602
05251605
05251609
05251603
05251606
05251610
05251604
05251607
05251611
change per minute
Count
Count
Count
